# Supplementary material for: Novel 3,19-(N-Phenyl-3-(4-fluorophenyl)-pyrazole) Acetal of Andrographolide Promotes Cell Cycle Arrest and Apoptosis in MDA-MB-231 Breast Cancer Cells
Source: Pharmaceuticals (Basel). 2025 Jul 10;18(7):1026. doi: 10.3390/ph18071026 (PMC12298351; doi:10.3390/ph18071026)
Supplement: Supplementary file 1 [file pharmaceuticals-18-01026-s001.zip › pharmaceuticals-3665412-supplementary.pdf]

Novel 3,19-(N-phenyl-3-(4-fluorophenyl)-pyrazole) Acetal of Andrographolide Promotes Cell Cycle Arrest and Apoptosis in MD-MBA-231 Breast Cancer Cells

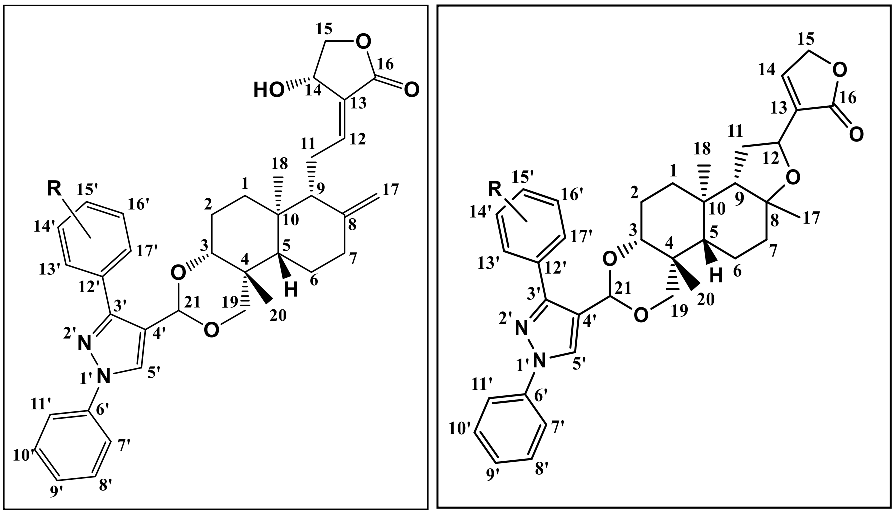

General structures and numbering of 3,19-(N-phenyl-3-aryl-pyrazole) acetals of andrographolide and 3,19-(N-phenyl-3-aryl-pyrazole) acetals of isoandrographolide.

Characterization

The UV-Vis spectra exhibited bands between 275 to 280 nm for all the synthesized compounds. In the IR spectra of 3,19-(N-phenyl-3-aryl-pyrazole) acetals of andrographolide peaks at ~3400 cm^{-1} and ~1672 cm^{-1} were observed for –OH and exocyclic C=C groups respectively. These peaks were absent for 3,19-(N-phenyl-3-aryl-pyrazole) acetals of isoandrographolide. The presence of the C-H stretching band within the 3089-3070 cm^{-1} range and the aromatic skeletal bands spanning from 1600 to 1450 cm^{-1}, confirm the existence of the aromatic group. The band situated at 2948-2920 cm^{-1} corresponds to the sp^3 C-H stretch originating from the diterpene moiety. The peak between the range 1732-1759 cm^{-1} corresponds to the carbonyl group (C=O). Distinct absorptions at approximately 1220 and 1100 cm^{-1} can be attributed to the C-O stretching. Compounds with aryl chloride have a band at 1071-1060 cm^{-1} (Ar-Cl stretch). Compounds with aryl fluorides have a band around 1245 - 1220 cm^{-1} (Ar-F stretch) and compounds with aryl bromides have a band between 1020-1011 cm^{-1}(Ar-Br stretch). Furthermore, the C-H out-of-plane bending vibrations of the aromatic ring were observed within the 890 to 750 cm^{-1} range.

In the ^{1}H NMR spectra of all the acetals, the peak at ~ δ 8.18 is due to the presence of H-5' (only hydrogen in the pyrazole ring) whereas peaks around δ 6.99 to 8.76 correspond to the aromatic protons attached to the pyrazole ring (H-7' to H-17'). The proton of unsaturated lactone (H-14) of ISOADG was observed at ~ δ 7.29. The peak at ~ δ 6.99 corresponds to H-12 of ADG. For all the compounds, a singlet peak at ~ δ 5.87 appeared for H-21. H-19, H-15 and H-12 protons in the case of 3,19-(N-phenyl-3-aryl-pyrazole) acetals of isoandrographolide and H-19, H-17, H-15, H-14 and H-12 protons for 3,19-(N-phenyl-3-aryl-pyrazole) acetals of andrographolide were deshielded and appeared around δ 5.00-3.61. For 3,19-(N-phenyl-3-aryl-pyrazole) acetals of isoandrographolide, protons (H-17, H-18 and H-20) of the -CH_3 groups were identified by three singlets in the range of ~ δ 1.50-1.11. While for 3,19-(N-phenyl-3-aryl-pyrazole) acetals of andrographolide, protons (H-18 and H-20) of the -CH_3 group were identified by two singlets at ~ δ 1.50 and ~ δ 0.85. The -OCH_3 (H-18') and -CH_3 (H-18') substituted derivatives showed singlets at ~ δ 3.86 and δ 2.41 respectively.

In the ^{13}C NMR spectra, signals in the range of δ 173-170, indicate the presence of a carbonyl group (C=O). As anticipated, the carbons in the pyrazole ring and aromatic ring carbons were identified in the ~ δ 163 to 115 ppm range. In 3,19-(N-phenyl-3-aryl-pyrazole) acetals of andrographolide, C-8 and C-17 (exocyclic C=C) signals were observed at ~ δ 148 and δ 110 whereas in 3,19-(N-phenyl-3-aryl-pyrazole) acetals of isoandrographolide they were seen at ~ δ 82 and δ 31. Notably, the signal around δ 90 attributed to C-21 bonded to two oxygen atoms provides strong evidence for the formation of acetals. In the diterpinoid part carbon atoms linked to oxygen atoms (-O-C) were detected in the δ 82 to 66 region. Additionally, consistent with expectations, signals associated with –C, –CH, –CH_2, and –CH_3 groups were observed in the δ 59 to 16 range. Peaks corresponding to the –OCH_3 group were discernible at around δ 55.

3,19-(N-phenyl-3-(phenyl)-pyrazole) acetal of andrographolide (1a):

White solid, yield 90%, m.p. 260.1-261.3°C; **UV (λ_{max})**: 275 nm; **IR (KBr) cm^{-1}:** 3390 (O-H stretch), 3060 (aromatic C-H stretch), 2940 (sp^3 C-H stretch), 1732 (C=O stretch), 1673 (exocyclic C=C stretch), 1600 (conjugated C=C stretch), 1460 (aromatic C=C stretch), 1219 and 1103 (C-O stretch), 892 & 759 (aromatic C-H out of plane bending); **^{1}H NMR (400 MHz, $CDCl_3$)**: δ 8.20 (1H, s, H-5'), 7.82 (2H, d, J=8.40 Hz, H-13' and H-17'), 7.75 (2H, d, J=7.68 Hz, H-7' and H-11'), 7.45 (2H, t, J=7.72 Hz, H-14' and H-16'), 7.41-7.38 (3H, m, H-8', H-10', H-15'), 7.29-7.28 (1H, m, H-9'), 6.95 (1H, dt, J=6.4 Hz, 1.48 Hz, H-12), 5.91 (1H, s, H-21), 4.99 (1H, d, J=5.64 Hz, H-14), 4.91 (1H, s, H_a-17), 4.61 (1H, s, H_b-17), 4.41 (1H, dd, J=10.48 Hz, 6.12 Hz, H_a-15), 4.22 (1H, d, J= 11.52 Hz, H_b-15), 4.26 (1H, m, H_a-19), 3.70 (1H, m, H-3), 3.60 (1H, m, H_b-19), 2.54 (1H, m, H_a-11), 2.46-2.38 (1H, m, H_b-11), 2.07-2.00 (1H, m, H_a-1), 1.85 (3H, m, H_a-7, H-2), 1.63 (4H, m, H_b-1, H_a-6, H-9, H_b-6), 1.51 (3H, s, H-18), 1.30-1.25 (3H, m), 0.82 (3H, s, H-20); **^{13}C NMR (100 MHz, $CDCl_3$)**: δ 169.92 (C-16), δ 150.77 (C-3'), δ 148.78 (C-8), δ 146.38 (C-12), δ 139.95 (C-6'), δ 133.00 (C-12'), δ 129.38 (C-8' and C-10'), δ 128.47 (C-14' and C-16'), δ 128.25 (C-13' and C-17'), δ 128.13 (C-15'), δ 127.98 (C-13), δ 127.19 (C-9'), δ 126.63 (C-5'), δ 120.43 (C-4'), δ 119.31 (C-7' and C-11'), δ 109.31 (C-17), δ 90.21 (C-21), δ 80.96 (C-3), δ 74.29 (C-15), δ 69.57 (C-19), δ 66.16 (C-14), δ 55.71 (C-9), δ 54.76 (C-5), δ 38.84 (C-4), δ 37.55 (C-7), δ 36.85 (C-10), δ 36.04 (C-1), δ 26.11 (C-2), δ 24.72 (C-6), δ 22.78 (C-11), δ 21.65 (C-20), δ 15.35 (C-18); **HRMS (*m/z*):** Found $[M+H]^+$ = m/z 581.3001, calculated $[M]^+$ = m/z 580.2937

3,19-(N-phenyl-3-(3-nitrophenyl)-pyrazole) acetal of andrographolide (1b):

White solid, yield 87%, m.p. 125.5-127.8°C; **UV (λ_{max})**: 275 nm; **IR (KBr) cm^{-1}:** 3428 (O-H stretch), 3077 (aromatic C-H stretch), 2932 (sp^3 C-H stretch), 1737 (C=O stretch), 1672 (exocyclic C=C stretch), 1601 (conjugated C=C stretch), 1536 (N-O stretching), 1460 (aromatic C=C stretch), 1215 and 1098 (C-O stretch), 900 & 760 (aromatic C-H out of plane bending); **^{1}H NMR (400 MHz, $CDCl_3$)**: δ 8.72 (1H, t, J= 1.76 Hz, H-13'), δ 8.28 (1H, d, J= 7.80 Hz, H-15'), δ 8.23 (1H, dd, J= 7.40 Hz, 1.40 Hz, H-17'), δ 8.21 (1H, s, H-5'), 7.76 (2H, d, J= 7.84 Hz, H-7' and H-11'), 7.61 (1H, t, J= 8.0 Hz, H-16'), 7.47 (2H, t, J= 7.64 Hz, H-8' and H-10'), 7.32 (1H, t, J= 7.44 Hz, H-9'), 6.95 (1H, t, J=6.72 Hz, H-12), 5.99 (1H, s, H-21), 5.05 (1H, d, J=5.72 Hz, H-14), 4.92 (1H, s, H_a-17), 4.63 (1H, s, H_b-17), 4.46 (1H, dd, J=10.48 Hz, 6.12 Hz, H_a-15), 4.29 (1H, d, J= 11.52 Hz, H_b-15), 4.26 (1H, dd, J= 10.48 Hz, 2.0 Hz, H_a-19), 3.73 (1H, dd, J=12.32 Hz, 4.20 Hz, H-3), 3.64 (1H, d, J=11.40 Hz, H_b-19), 2.60-2.41 (5H,

m), 2.02-1.86 (1H, m), 1.91-1.87 (4H, m), 1.49 (3H, s, H-18), 1.33-1.25 (3H, m), 0.85 (3H, s, H-20); **^{13}C NMR (100 MHz, $CDCl_3$)**: δ 170.03 (C-16), δ 148.69 (C-3'), δ 148.40 (C-14'), δ 148.01 (C-8), δ 146.34 (C-12), δ 139.71 (C-6'), δ 134.83 (C-12'), δ 134.03 (C-17'), δ 129.50 (C-8' and C-10'), δ 129.44 (C-13'), δ 128.08 (C-13), δ 127.82 (C-9'), δ 127.06 (C-5'), δ 122.95 (C-15'), δ 122.79 (C-16'), δ 120.92 (C-4'), δ 119.36 (C-7' and C-11'), δ 109.37 (C-17), δ 89.93 (C-21), δ 81.11 (C-3), δ 74.37 (C-15), δ 69.53 (C-19), δ 66.18 (C-14), δ 55.74 (C-9), δ 54.81 (C-5), δ 38.85 (C-4), δ 37.57 (C-7), δ 36.89 (C-10), δ 36.08 (C-1), δ 26.08 (C-2), δ 24.72 (C-6), δ 22.80 (C-11), δ 21.54 (C-20), δ 15.37 (C-18); **HRMS (*m/z*):** Found $[M+H]^+$ = m/z 626.2859, calculated $[M]^+$ = m/z 625.2788.

3,19-(N-phenyl-3-(3-fluorophenyl)-pyrazole) acetal of andrographolide (1c):

White solid, yield 88%, m.p. 142.2-144.0°C; **UV (λ_{max})**: 275 nm; **IR (KBr) cm^{-1}:** 3421 (O-H stretch), 3073 (aromatic C-H stretch), 2940 (sp^3 C-H stretch), 1742 (C=O stretch), 1673 (exocyclic C=C stretch), 1600 (conjugated C=C stretch), 1463 (aromatic C=C stretch), 1245 (aromatic C-F stretch), 1216 and 1100 (C-O stretch), 895 & 759 (aromatic C-H out of plane bending); **^{1}H NMR (400 MHz, $CDCl_3$)**: δ 8.19 (1H, s, H-5'), 7.74 (2H, d, J=7.64 Hz, H-7' and H-11'), 7.64 (1H, d, J=7.80 Hz, H-17'), 7.60 (1H, d, J=10.16 Hz, H-13'), 7.45 (2H, t, J=7.60 Hz, H-8' and H-10'), 7.41 (1H, m, H-16'), 7.30 (1H, t, J=7.40 Hz, H-9'), 7.07 (1H, dt, J=8.44 Hz, J=1.92 Hz, H-15'), 6.95 (1H, dt, J=6.72 Hz, 1.46 Hz, H-12), 5.91 (1H, s, H-21), 5.01 (1H, d, J=5.76 Hz, H-14), 4.91 (1H, s, H_a-17), 4.62 (1H, s, H_b-17), 4.44 (1H, dd, J=10.48 Hz, 6.16 Hz, H_a-15), 4.25-4.23 (1H, m, H_b-15 H_a-19), 3.71 (1H, dd, J=12.64 Hz, 4.36 Hz, H-3), 3.61 (1H, d, J=11.04 Hz, H_b-19), 3.47 (2H, m), 2.62-2.37 (5H, m), 2.05-1.99 (1H, m), 1.91-1.87 (4H, m), 1.50 (3H, s, H-18), 1.31-1.26 (3H, m), 0.84 (3H, s, H-20); **^{13}C NMR (100 MHz, $CDCl_3$)**: δ 169.96 (C-16), δ 162.93 (C-14',d, J=243.36 Hz), δ 149.43 (C-3'), δ 148.77 (C-8), δ 146.38 (C-12), δ 139.85 (C-6'), δ 135.18 (C-12', d, J=7.78 Hz,), δ 129.99 (C-16',d, J=8.41 Hz,) δ 129.43 (C-8' and C-10'), δ 128.04 (C-13), δ 127.44 (C-9'), δ 126.81 (C-5'), δ 123.79 (C-17'), δ 120.58 (C-4'), δ 119.33 (C-7' and C-11'), δ 115.17 (C-13',d, J=22.78 Hz,), δ 114.92 (C-15',d, J=20.88 Hz), δ 109.36 (C-17), δ 90.08 (C-21), δ 81.05 (C-3), δ 74.33 (C-15), δ 69.58 (C-19), δ 66.20 (C-14), δ 55.74 (C-9), δ 54.79 (C-5), δ 38.86 (C-4), δ 37.57 (C-7), δ 36.87 (C-10), δ 36.07 (C-1), δ 26.11 (C-2), δ 24.75 (C-6), δ 22.80 (C-11), δ 21.58 (C-20), δ 15.39 (C-18); **HRMS (*m/z*):** Found $[M+H]^+$ = m/z 599.2861, calculated $[M]^+$ = m/z 598.2843.

3,19-(N-phenyl-3-(3-chlorophenyl)-pyrazole) acetal of andrographolide (1d)

White solid, yield 91%, m.p. 143.2-145.1°C; **UV (λ_{max})**: 275 nm; **IR (KBr) cm^{-1}:** 3425 (O-H stretch), 3073 (aromatic C-H stretch), 2941 (sp^3 C-H stretch), 1756 (C=O stretch), 1673 (exocyclic C=C stretch), 1600 (conjugated C=C stretch), 1454 (aromatic C=C stretch), 1216 and 1101 (C-O stretch), 1071 (aromatic C-Cl stretch), 900 & 756 (aromatic C-H out of plane bending); **^{1}H NMR (400 MHz, $CDCl_3$)**: δ 8.18 (1H, s, H-5'), 7.88 (1H, t, J=1.84 Hz, H-13'), 7.76-7.73 (3H, m, H-15', H-7' and H-11'), 7.45 (2H, t, J=7.52 Hz, H-8' and H-10'), 7.40-7.35 (2H, m, H-16' and H-17'), 7.30 (1H, t, J=7.40 Hz, H-9'), 6.95 (1H, dt, J=6.72 Hz, 1.44 Hz, H-12), 5.91 (1H, s, H-21), 5.02 (1H, d, J=5.68 Hz, H-14), 4.91 (1H, s, H_a-17), 4.62 (1H, s, H_b-17), 4.44 (1H, dd, J=10.44 Hz, 6.12 Hz, H_a-15), 4.25-4.22 (2H, d, m, H_b-15, H_a-19), 3.71 (1H, dd, J=12.52 Hz, 4.32 Hz, H-3), 3.61 (1H, d, J=11.32, H_b-19), 3.47 (2H, m), 2.62-2.37 (5H, m), 2.07-1.85 (1H, m), 1.89-1.85 (4H, m), 1.50 (3H, s, H-18), 1.31-1.26 (3H, m), 0.84 (3H, s, H-20); **^{13}C NMR (100 MHz, $CDCl_3$)**: δ 169.93 (C-16), δ 149.25 (C-3'), δ 148.77 (C-8), δ 146.37 (C-12), δ 139.84 (C-6'), δ 134.80 (C-14'), δ 134.36 (C-12'), δ 129.77 (C-13') δ 129.44 (C-8' and C-10'), δ 128.26 (C-16'), δ 128.10 (C-15'), δ 128.05 (C-13), δ 127.43 (C-9'), δ 126.83 (C-5'), δ 126.22 (C-17'), δ 120.59 (C-4'), δ 119.34 (C-7' and C-11'), δ 109.35 (C-17), δ 90.09 (C-21), δ 81.08 (C-3), δ 74.32 (C-15), δ 69.60 (C-19), δ 66.21 (C-14), δ 55.74 (C-9), δ 54.78 (C-5), δ 38.86 (C-4), δ 37.57 (C-7), δ 36.87 (C-10), δ 36.08 (C-1), δ 26.11 (C-2), δ 24.74 (C-6), δ 22.80 (C-11), δ 21.61 (C-20), δ 15.37 (C-18); **HRMS (*m/z*):** Found $[M+H]^+$ = m/z 615.2553, $[M+H+2]^+$ = m/z 617.2553, calculated $[M]^+$ = m/z 614.2548.

3,19-(N-phenyl-3-(3-bromophenyl)-pyrazole) acetal of andrographolide (1e):

White solid, yield 87%, m.p. 234.0-236.1°C; **UV (λ_{max})**: 275 nm; **IR (KBr) cm^{-1}:** 3366 (O-H stretch), 3084 (aromatic C-H stretch), 2935 (sp^3 C-H stretch), 1739 (C=O stretch), 1674 (exocyclic C=C stretch), 1599 (conjugated C=C stretch), 1456 (aromatic C=C stretch), 1217 and 1102 (C-O stretch), 1019 (Ar-Br stretch), 889 & 759 (aromatic C-H out of plane bending); **^{1}H NMR (400 MHz, $CDCl_3$)**: δ 8.18 (1H, s, H-5'), 8.02 (1H, t, J=1.64 Hz, H-13'), 7.80 (1H, d, J=7.78 Hz, H-15'), 7.74 (2H, d, J=7.60 Hz, H-7', H-11'), 7.51 (1H, d, J=7.80 Hz, H-17'), 7.45 (2H, t, J=7.56 Hz, H-8'and H-10'), 7.32 (1H, t, J=7.80 Hz, H-9'), 7.30 (1H, m, H-16'), 6.95 (1H, dt, J=6.80 Hz, 1.36 Hz, H-12), 5.90 (1H, s, H-21), 5.11 (1H, s, H-14), 4.91 (1H, s, H_a-17), 4.62 (1H, s, H_b-17), 4.44 (1H, dd, J=10.48 Hz, 6.12 Hz, H_a-15), 4.25-4.22 (2H, m, H_b-15, H_a-19), 3.71 (1H, dd, J=12.52 Hz, 4.32 Hz, H-3), 3.61 (1H, d, J=11.28, H_b-19), 3.47 (2H, m), 2.63-2.38 (5H, m), 2.05-1.93 (1H, m), 1.89-1.85 (4H, m), 1.50 (3H, s, H-18), 1.31-1.25

(3H, m), 0.84 (3H, s, H-20); **^{13}C NMR (100 MHz, CDCl$_3$)**: δ 169.94 (C-16), δ 149.12 (C-3'), δ 148.77 (C-8), δ 146.36 (C-12), δ 139.83 (C-6'), δ 135.06 (C-12'), δ 131.09 (C-16'), δ 131.02 (C-13'), δ 130.06 (C-15'), δ 129.45 (C-8'), δ 128.05 (C-13), δ 127.43 (C-9'), δ 126.84 (C-5'), δ 126.69 (C-17'), δ 122.58 (C-14'), δ 120.59 (C-4'), δ 119.35 (C-7'), δ 109.36 (C-17), δ 90.07 (C-21), δ 81.08 (C-3), δ 74.33 (C-15), δ 69.60 (C-19), δ 66.21 (C-14), δ 55.74 (C-9), δ 54.77 (C-5), δ 38.86 (C-4), δ 37.57 (C-7), δ 36.86 (C-10), δ 36.06 (C-1), δ 26.12 (C-2), δ 24.73 (C-6), δ 22.80 (C-11), δ 21.67 (C-20), δ 15.37 (C-18); **HRMS (*m/z*):** $[M+H]^+$ = m/z 659.2051, $[M+H+2]^+$ = m/z 661.2051, calculated $[M]^+$ = m/z 658.2042.

3,19-(N-phenyl-3-(4-fluorophenyl)-pyrazole) acetal of andrographolide (1f):

White solid, yield 92%, m.p. 260.3-262.5°C; **UV (λ_{max})**: 275 nm; **IR (KBr) cm^{-1}:** 3366 (O-H stretch), 3083 (aromatic C-H stretch), 2930 (sp^3 C-H stretch), 1735 (C=O stretch), 1673 (exocyclic C=C stretch), 1602 (conjugated C=C stretch), 1462 (aromatic C=C stretch), 1220 and 1104 (C-O stretch), 989, 889 & 758 (aromatic C-H out of plane bending). **^{1}H NMR (400 MHz, CDCl$_3$)**: δ 8.18 (1H, s, H-5'), 7.82-7.78 (2H, m, H-13' and H-17'), 7.73 (2H, d, J=7.68 Hz, H-7' and H-11') 7.44 (2H, t, J=7.68 Hz, H-8' and H-10'), 7.29 (1H, t, J=7.44 Hz, H-9'), 7.16-7.12 (2H, t, J=8.72 Hz, H-14' and H-16'), 6.94 (1H, t, J=6.64 Hz, H-12), 5.87 (1H, s, H-21), 5.00 (1H, s, H-14), 4.91 (1H, s, H_a-17), 4.62 (1H, s, H_b-17), 4.43 (1H, dd, J=10.44 Hz, 6.12 Hz, H_a-15), 4.23 (2H, m, H_b-15, H_a-19), 3.70 (1H, dd, J=12.84 Hz, 4.76 Hz, H-3), 3.60 (1H, d, J=11.36, H_b-19), 2.56-2.52 (2H, m), 2.04-1.98 (1H, m), 1.90-1.81 (4H, m), 1.50 (3H, s, H-18), 1.31-1.25 (3H, m), 0.83 (3H, s, H-20). **^{13}C NMR (100 MHz, CDCl$_3$)**: δ 170.03 (C-16), δ 163.03 (d, C-15', J=245.69 Hz,), δ 149.89 (C-3'), δ 148.83 (C-8), δ 146.37 (C-12), δ 139.89 (C-6'), δ 130.03 (d, C-13' and C-17', J=8.02 Hz,), δ 129.43 (C-8' and C-10'), δ 129.15 (d, C-12', J=2.99 Hz), δ 128.01 (C-13), δ 127.29 (C-5'), δ 126.73 (C-9'), δ 120.30 (C-4'), δ 119.31 (C-7' and C-11'), δ 115.43 (d, C-14' and C-16', J=21.35 Hz,) δ 109.39 (C-17), δ 90.16 (C-21), δ 80.96 (C-3), δ 74.36 (C-15), δ 69.59 (C-19), δ 66.19 (C-14), δ 55.72 (C-9), δ 54.79 (C-5), δ 38.85 (C-4), δ 37.56 (C-7), δ 36.86 (C-10), δ 36.03 (C-1), δ 26.12 (C-2), δ 24.76 (C-6), δ 22.79 (C-11), δ 21.66 (C-20), δ 15.40 (C-18); **HRMS (*m/z*):** Found $[M+H]^+$ = m/z 599.2873, calculated $[M]^+$ = m/z 598.2843.

3,19-(N-phenyl-3-(4-chlorophenyl)-pyrazole) acetal of andrographolide (1g):

White solid, yield 86%, m.p. 142.3-144.5°C; **UV (λ_{max})**: 280 nm; **IR (KBr) cm^{-1}:** 3434 (O-H stretch), 3075 (aromatic C-H stretch), 2938 (sp^3 C-H stretch), 1755 (C=O stretch), 1673 (exocyclic C=C stretch), 1601 (conjugated C=C stretch), 1460 (aromatic C=C stretch), 1218

and 1100 (C-O stretch), 1069 (aromatic C-Cl stretch), 875 & 760 (aromatic C-H out of plane bending); **^{1}H NMR (400 MHz, $CDCl_3$)**: δ 8.18 (1H, s, H-5'), 7.77 (2H, d, J=8.48 Hz, H-13' and H-17'), 7.73 (2H, d, J=7.72 Hz, H-7' and H-11') 7.47-7.41 (4H, m, H-14', H-16', H-8' and H-10'), 7.30 (2H, t, J=7.44 Hz, H-9'), 6.95 (1H, dt, J=6.32 Hz, 1.36 Hz, H-12), 5.87 (1H, s, H-21), 5.01 (1H, d, J=6.0 Hz, H-14), 4.91 (1H, s, H_a-17), 4.62 (1H, s, H_b-17), 4.44 (1H, dd, J=10.44 Hz, 6.12 Hz, H_a-15), 4.25-4.21 (2H, m, H_b-15, H_a-19), 3.70 (1H, dd, J=12.88 Hz, 4.76 Hz, H-3), 3.60 (1H, d, J=11.36 Hz, H_b-19), 2.57-2.35 (5H, m), 2.04-1.99 (1H, m), 1.91-1.81 (4H, m), 1.50 (3H, s, H-18), 1.31-1.23 (3H, m), 0.83 (3H, s, H-20); **^{13}C NMR (100 MHz, $CDCl_3$)**: δ 170.02 (C-16), δ 149.63 (C-3'), δ 148.82 (C-8), δ 146.37 (C-12), δ 139.85 (C-6'), δ 134.11 (C-15'), δ 131.52 (C-12'), δ 129.53 (C-8' and C-10'), δ 129.45 (C-14' and C-16'), δ 128.67 (C-13'and C-17'), δ 128.03 (C-13), δ 127.41 (C-9'), δ 126.80 (C-5'), δ 120.45 (C-4'), δ 119.77 (C-7' and C-11'), δ 109.40 (C-17), δ 90.11 (C-21), δ 80.98 (C-3), δ 74.37 (C-15), δ 69.60 (C-19), δ 66.20 (C-14), δ 55.73 (C-9), δ 54.76 (C-5), δ 38.85 (C-4), δ 37.56 (C-7), δ 36.87 (C-10), δ 36.04 (C-1), δ 26.12 (C-2), δ 24.77 (C-6), δ 22.79 (C-11), δ 21.66 (C-20), δ 15.40 (C-18); **HRMS (*m/z*):** Found $[M+H]^+$ = m/z 615.2556, $[M+H+2]^+$ = m/z 617.2556, calculated $[M]^+$ = m/z 614.2548.

3,19-(N-phenyl-3-(4-bromophenyl)-pyrazole) acetal of andrographolide (1h):

White solid, yield 83%, m.p. 135.0-137.6°C; **UV (λ_{max})**: 280 nm; **IR (KBr) cm^{-1}:** 3427 (O-H stretch), 3081 (aromatic C-H stretch), 2943 (sp^3 C-H stretch), 1755 (C=O stretch), 1672 (exocyclic C=C stretch), 1601 (conjugated C=C stretch), 1458 (aromatic C=C stretch), 1220 and 1101 (C-O stretch), 1011 (aromatic C-Br stretch), 872 & 757 (aromatic C-H out of plane bending); **^{1}H NMR (400 MHz, $CDCl_3$)**: δ 8.18 (1H, s, H-5'), 7.74-7.70 (4H, m, H-13', H-17', H-7' and H-11'), 7.57 (2H, d, J=8.44 Hz, H-14' and H-16'), 7.45 (2H, t, J=7.64 Hz, H-8' and H-10'), 7.30 (1H, t, J=7.40 Hz, H-9'), 6.95 (1H, dt, J=7.0 Hz, 1.32 Hz, H-12), 5.87 (1H, s, H-21), 5.02 (1H, d, J=6.0 Hz, H-14), 4.91 (1H, s, H_a-17), 4.62 (1H, s, H_b-17), 4.45 (1H, dd, J=10.48 Hz, 6.16 Hz, H_a-15), 4.25-4.21 (2H, m, H_b-15, H_a-19), 3.70 (1H, dd, J=12.88 Hz, 4.76 Hz, H-3), 3.60 (1H, d, J=11.40, H_b-19), 2.58-2.37 (5H, m), 2.04-1.99 (1H, m), 1.87-1.81 (4H, m), 1.50 (3H, s, H-18), 1.31-1.23 (3H, m), 0.83 (3H, s, H-20). **^{13}C NMR (100 MHz, $CDCl_3$)**: δ 170.01 (C-16), δ 149.63 (C-3'), δ 148.82 (C-8), δ 146.37 (C-12), δ 139.85 (C-6'), δ 131.98 (C-12'), δ 131.61 (C-14' and C-16'), δ 129.83 (C-8' and C-10'), δ 129.45 (C-13' and C-17'), δ 128.02 (C-13), δ 127.43 (C-9'), δ 126.82 (C-5'), δ 122.38 (C-15'), δ 120.45 (C-4'), δ 119.35 (C-7' and C-11'), δ 109.40 (C-17), δ 90.11 (C-21), δ 80.98 (C-3), δ 74.37 (C-15), δ 69.60 (C-19), δ 66.20 (C-14), δ 55.73 (C-9), δ 54.76 (C-5), δ 38.86 (C-4), δ 37.58 (C-7), δ 36.86 (C-10),

δ 36.04 (C-1), δ 26.12 (C-2), δ 24.77 (C-6), δ 22.79 (C-11), δ 21.67 (C-20), δ 15.41 (C-18); **HRMS (*m/z*):** Found $[M+H]^+$ = m/z 659.2031, $[M+H+2]^+$ = m/z 661.2031, calculated $[M]^+$ = m/z 658.2042.

3,19-(N-phenyl-3-(4-methylphenyl)-pyrazole) acetal of andrographolide (1i):

White solid, yield 90%, m.p. 143.2-145.5°C; **UV (λ_{max})**: 275 nm; **IR (KBr) cm^{-1}:** 3430 (O-H stretch), 3078 (aromatic C-H stretch), 2943 (sp^3 C-H stretch), 1758 (C=O stretch), 1672 (exocyclic C=C stretch), 1600 (conjugated C=C stretch), 1463 (aromatic C=C stretch), 1221 and 1101 (C-O stretch), 890 & 757 (aromatic C-H out of plane bending); **^{1}H NMR (400 MHz, $CDCl_3$)**: δ 8.19 (1H, s, H-5'), 7.74 (2H, d, J=7.72 Hz, H-7' and H-11'), 7.71 (2H, d, J=8.04 Hz, H-13' and 17'), 7.44 (1H, t, J=7.64 Hz, H-9'), 7.28 (4H, m, H-8', H-10', H-14' and H-16'), 6.94 (1H, t, J=6.72 Hz, H-12), 5.90 (1H, s, H-21), 4.99 (1H, d, J=5.96 Hz, H-14), 4.91 (1H, s, H_a-17), 4.61 (1H, s, H_b-17), 4.43 (1H, dd, J=10.48 Hz, 6.12 Hz, H_a-15), 4.21 (2H, m, H_b-15, H_a-19), 3.70 (1H, dd, J=12.48 Hz, J=4.60 Hz, H-3), 3.59 (1H, d, J=11.32, H_b-19), 2.56-2.51 (2H, m), 2.41 (3H, s, H-18'), 2.04-1.97 (1H, m), 1.86-1.83 (4H, m), 1.51 (3H, s, H-18), 1.28 (3H, m), 0.81 (3H, s, H-20); **^{13}C NMR (100 MHz, $CDCl_3$)**: δ 170.02 (C-16), δ 150.87 (C-3'), δ 148.82 (C-8), δ 146.41 (C-12), δ 140.00 (C-6'), δ 137.93 (C-15'), δ 130.11 (C-12'), δ 129.39 (C-14' and C-16'), δ 129.23 (C-8' and C-10'), δ 128.15 (C-13' and C-17'), δ 128.00 (C-13), δ 127.14 (C-9'), δ 126.58 (C-5'), δ 120.29 (C-4'), δ 119.33 (C-7' and C-11'), δ 109.33 (C-17), δ 90.27 (C-21), δ 80.98 (C-3), δ 74.35 (C-15), δ 69.60 (C-19), δ 66.16 (C-14), δ 55.73 (C-9), δ 54.77 (C-5), δ 38.85 (C-4), δ 37.58 (C-7), δ 36.87 (C-10), δ 36.05 (C-1), δ 26.13 (C-2), δ 24.74 (C-6), δ 22.80 (C-11), δ 21.68 (C-20), δ 15.36 (C-18); **HRMS (*m/z*):** Found $[M+H]^+$ = m/z 595.3112, calculated $[M]^+$ = m/z 594.3094.

3,19-(N-phenyl-3-(4-methoxyphenyl)-pyrazole) acetal of andrographolide (1j):

White solid, yield 93%, m.p. 145.2-148.3°C; **UV (λ_{max})**: 280 nm; **IR (KBr) cm^{-1}:** 3435 (O-H stretch), 3078 (aromatic C-H stretch), 2920 (sp^3 C-H stretch), 1759 (C=O stretch), 1673 (exocyclic C=C stretch), 1600 (conjugated C=C stretch), 1462 (aromatic C=C stretch), 1250, 1220 and 1101 (C-O stretch), 890 & 757 (aromatic C-H out of plane bending); **^{1}H NMR (400 MHz, $CDCl_3$)**: δ 8.17 (1H, s, H-5'), 7.75 (2H, d, J=7.61 Hz, H-7' and H-11'), 7.73 (2H, d, J=8.76 Hz, H-13' and H-17'), 7.43 (2H, t, J=7.60 Hz, H-8' and H-10'), 7.28 (1H, t, J=7.4 Hz, H-9'), 6.99 (2H, d, J=8.80 Hz, H-14' and H-16'), 6.94 (1H, dt, J=6.92 Hz, 1.48 Hz, H-12), 5.88 (1H, s, H-21), 4.98 (1H, s, H-14), 4.91 (1H, s, H_a-17), 4.61 (1H, s, H_b-17), 4.42 (1H, dd, J=10.44 Hz, 6.12 Hz, H_a-15), 4.22 (2H, m, H_b-15, H_a-19), 3.86 (3H, s, H-18') 3.69 (1H, dd, J=12.84

Hz, 4.68 Hz, H-3), 3.59 (1H, d, J=11.36 Hz, H_b-19), 2.63-2.35 (6H, m), 2.04-1.98 (1H, m), 1.89-1.84 (4H, m), 1.50 (3H, s, H-18), 1.30-1.25 (3H, m), 0.82 (3H, s, H-20); **^{13}C NMR (100 MHz, $CDCl_3$)**: δ 169.98 (C-16), δ 159.67 (C-15'), δ 150.64 (C-3'), δ 148.77 (C-8), δ 146.42 (C-12), δ 140.01 (C-6'), δ 129.52 (C-8' and C-10'), δ 129.38 (C-13' and C-17'), δ 128.01 (C-13), δ 127.08 (C-9'), δ 126.52 (C-5'), δ 125.62 (C-12'), δ 120.12 (C-4'), δ 119.26 (C-7' and C-11'), δ 113.92 (C-14' and C-16'), δ 109.32 (C-17), δ 90.30 (C-21), δ 80.96 (C-3), δ 74.33 (C-15), δ 69.59 (C-19), δ 66.16 (C-14), δ 55.74 (C-9), δ 55.35 (C-18'), δ 54.78 (C-5), δ 38.86 (C-4), δ 37.58 (C-7), δ 36.87 (C-10), δ 36.06 (C-1), δ 26.13 (C-2), δ 24.75 (C-6), δ 22.80 (C-11), δ 21.68 (C-20), δ 15.37 (C-18); **HRMS (*m/z*):** Found $[M+H]^+$ = m/z 611.3065, calculated $[M]^+$ = m/z 610.2041.

3,19-(N-phenyl-3-(phenyl)-pyrazole) acetal of isoandrographolide (2a):

White solid, yield 85%, m.p. 218.2-220.3°C; **UV (λ_{max})**: 275 nm; **IR (KBr) cm^{-1}:** 3073 (aromatic C-H stretch), 2945 (sp^3 C-H stretch), 1755 (C=O stretch), 1601 (conjugated C=C stretch), 1460 (aromatic C=C stretch), 1202 and 1098 (C-O stretch), 887 & 755 (aromatic C-H out of plane bending); **^{1}H NMR (400 MHz, $CDCl_3$)**: δ 8.20 (1H, s, H-5'), 7.83 (2H, d, J=8.40 Hz, H-13' and H-17'), 7.76 (2H, d, J=7.68 Hz, H-7' and H-11'), 7.46 (2H, t, J=7.72 Hz, H-8' and H-10'), 7.44-7.37 (3H, m, H-14', H-15', H-16'), 7.30-7.28 (2H, m, H-9', H-14), 5.92 (1H, s, H-21), 4.81 (1H, s, H-15), 4.70 (1H, dt, J=9.28 Hz, 1.88 Hz, H-12), 4.33 (1H, d, J=11.48 Hz, H_a-19), 3.69 (1H, bd, J=12.64 Hz, H-3), 3.65 (1H, d, J=11.84 Hz, H_b-19), 2.46 (1H, m, H_a-11), 2.37 (1H, m, H_b-11), 2.06-1.99 (1H, m, H_a-1), 1.88-1.73 (3H, m, H_a-7, H-2), 1.61-1.56 (4H, m, H_b-1, H_a-6, H-9, H_b-6), 1.51 (3H, s, H-18), 1.13 (3H, s, H-17), 1.10 (3H, s, H-20), 1.05 -1.01 (2H, m, H-5, H_b-7); **^{13}C NMR (100 MHz, $CDCl_3$)**: δ 172.58 (C-16), δ 150.82 (C-3'), δ 143.24 (C-14), δ 140.05 (C-6'), δ 138.35 (C-13), δ 133.04 (C-12'), δ 129.35 (C-8' and C-10'), δ 128.50 (C-14' and C-16'), δ 128.24 (C-13' and C-17'), δ 128.14 (C-15'), δ 127.14 (C-9'), δ 126.53 (C-5'), δ 120.53 (C-4'), δ 119.29 (C-7' and C-11'), δ 90.42 (C-21), δ 82.74 (C-8), δ 81.70 (C-3), δ 73.07 (C-12), δ 70.54 (C-15), δ 69.81 (C-19), δ 57.98 (C-9), δ 51.69 (C-5), δ 38.21 (C-4), δ 36.42 (C-7), δ 35.93 (C-10), δ 35.65 (C-1), δ 32.77 (C-11), δ 31.79 (C-17), δ 26.04 (C-18), δ 20.97 (C-2), δ 17.34 (C-6), δ 16.42 (C-20); **HRMS (*m/z*):** Found $[M+H]^+$ = m/z 581.3006, calculated $[M]^+$ = m/z 580.2937.

3,19-(N-phenyl-3-(3-nitrophenyl)-pyrazole) acetal of isoandrographolide (2b):

White solid, yield 82%, m.p. 238.0-240.2°C; **UV (λ_{max})**: 275 nm; **IR (KBr) cm^{-1}:** 3074 (aromatic C-H stretch), 2948 (sp^3 C-H stretch), 1753 (C=O stretch), 1601 (conjugated C=C stretch), 1537 (N-O stretching), 1462 (aromatic C=C stretch), 1200 and 1095 (C-O stretch), 890 & 758 (aromatic C-H out of plane bending); **^{1}H NMR (400 MHz, $CDCl_3$)**: δ 8.76 (1H, t, J= 1.88 Hz, H-13'), δ 8.25 (1H, d, J= 7.96 Hz, H-15'), δ 8.22 (1H, dd, J= 7.96 Hz, 1.44 Hz, H-17'), δ 8.21 (1H, s, H-5'), 7.76 (2H, d, J= 7.64 Hz, H-7' and H-11'), 7.61 (1H, dt, J= 8.0 Hz, H-16'), 7.47 (2H, t, J= 7.64 Hz, H-8' and H-10'), 7.32 (1H, t, J= 7.44 Hz, H-9'), 7.29 (1H, d, J= 1.72 Hz, H-14), 5.91 (1H, s, H-21), 4.81 (2H, bs, H-15), 4.70 (1H, dt, J=9.16 Hz, 1.92 Hz, H-12), 4.39 (1H, d, J=11.56 Hz, H_a-19), 3.71 (1H, dd, J=12.52 Hz, 4.42 Hz, H-3), 3.68 (1H, d, J=11.0 Hz, H_b-19), 2.47 (1H, m, H_a-11), 2.38 (1H, m, H_b-11), 2.07-2.00 (1H, m, H_a-1), 1.90-1.81 (3H, m, H_a-7, H-2), 1.62-1.57 (4H, m, H_b-1, H_a-6, H-9, H_b-6), 1.49 (3H, s, H-18), 1.13 (3H, s, H-17), 1.12 (3H, s, H-20), 1.05 -1.02 (2H, m, H-5, H_b-7); **^{13}C NMR (100 MHz, $CDCl_3$)**: δ 172.57 (C-16), δ 148.46 (C-3'), δ 148.15 (C-14'), δ 143.28 (C-14), δ 139.74 (C-6'), δ 138.32 (C-13), δ 134.85 (C-12'), δ 133.98 (C-17'), δ 129.48 (C-8' and C-10'), δ 129.37 (C-13'), δ 127.79 (C-9'), δ 126.99 (C-5'), δ 123.10 (C-15'), δ 122.78 (C-16'), δ 120.96 (C-4'), δ 119.34 (C-7' and C-11'), δ 90.13 (C-21), δ 82.72 (C-8), δ 81.79 (C-3), δ 73.06 (C-12), δ 70.54 (C-15), δ 69.74 (C-19), δ 57.98 (C-9), δ 51.69 (C-5), δ 38.21 (C-4), δ 36.43 (C-7), δ 35.91 (C-10), δ 35.63 (C-1), δ 32.76 (C-11), δ 31.77 (C-17), δ 26.00 (C-18), δ 20.86 (C-2), δ 17.34 (C-6), δ 16.41 (C-20); **HRMS (*m/z*):** Found $[M+H]^+$ = m/z 626.2852, calculated $[M]^+$ = m/z 625.2788.

3,19-(N-phenyl-3-(3-fluorophenyl)-pyrazole) acetal of isoandrographolide (2c):

White solid, yield 87%, m.p. 223.2-225.3°C; **UV (λ_{max})**: 275 nm; **IR (KBr) cm^{-1}:** 3074 (aromatic C-H stretch), 2948 (sp^3 C-H stretch), 1753 (C=O stretch), 1602 (conjugated C=C stretch), 1462 (aromatic C=C stretch), 1220 (aromatic C-F stretch), 1203 and 1102 (C-O stretch), 891 & 757 (aromatic C-H out of plane bending); **^{1}H NMR (400 MHz, $CDCl_3$)**: δ 8.19 (1H, s, H-5'), 7.75 (2H, d, J=7.56 Hz, H-7' and H-11'), 7.62 (1H, d, J=7.40 Hz, H-17'), 7.60 (1H, m, H-13'), 7.45 (2H, t, J=7.52 Hz, H-8' and H-10'), 7.41 (1H, m, J=7.92 Hz, H-16'), 7.31-7.26 (2H, m, H-9' and H-14), 7.08 (1H, t, J=8.32 Hz, H-15'), 5.92 (1H, s, H-21), 4.81 (1H, s, H-15), 4.70 (1H, dt, J=9.44 Hz, 2.0 Hz, H-12), 4.34 (1H, d, J=11.52 Hz, H_a-19), 3.70 (1H, dd, J=12.68 Hz, 4.64 Hz, H-3), 3.65 (1H, d, J=11.68 Hz, H_b-19), 2.47 (1H, m, H_a-11), 2.39 (1H, m, H_b-11), 2.07-1.99 (1H, m, H_a-1), 1.89-1.76 (3H, m, H_a-7, H-2), 1.61-1.56 (4H, m, H_b-1, H_a-

6, H-9, H_b-6), 1.50 (3H, s, H-18), 1.13 (3H, s, H-17), 1.11 (3H, s, H-20), 1.05 -1.02 (2H, m, H-5, H_b-7); **^{13}C NMR (100 MHz, $CDCl_3$)**: δ 172.54 (C-16), δ 162.94 (d, C-14', J=243.22 Hz), δ 149.48 (C-3'), δ 143.21 (C-14), δ 139.89 (C-6'), δ 138.34 (C-13), δ 135.18 (d, C-12', J=8.07 Hz), δ 129.95 (d, C-16', J=8.24 Hz) δ 129.38 (C-8' and C-10'), δ 127.36 (C-9'), δ 126.70 (C-5'), δ 123.76 (C-17'), δ 120.63 (C-4'), δ 119.28 (C-7' and C-11'), δ 115.16 (d, C-13', J=22.69 Hz,), δ 114.92 (d, C-15', J=21.14 Hz), δ 90.24 (C-21), δ 82.70 (C-8), δ 81.74 (C-3), δ 73.05 (C-12), δ 70.52 (C-15), δ 69.81 (C-19), δ 57.95 (C-9), δ 51.67 (C-5), δ 38.19 (C-4), δ 36.40 (C-7), δ 35.91 (C-10), δ 35.62 (C-1), δ 32.75 (C-11), δ 31.77 (C-17), δ 26.01 (C-18), δ 20.87 (C-2), δ 17.32 (C-6), δ 16.42 (C-20); **HRMS (*m/z*):** Found $[M+H]^+$ = m/z 599.2856, calculated $[M]^+$ = m/z 598.2843.

3,19-(N-phenyl-3-(3-chlorophenyl)-pyrazole) acetal of isoandrographolide (2d):

White solid, yield 85%, m.p. 225.3-227.1°C; **UV (λ_{max})**: 275 nm; **IR (KBr) cm^{-1}:** 3071 (aromatic C-H stretch), 2941 (sp^3 C-H stretch), 1757 (C=O stretch), 1600 (conjugated C=C stretch), 1453 (aromatic C=C stretch), 1060 (aromatic C-Cl stretch), 1205 and 1098 (C-O stretch), 885 & 756 (aromatic C-H out of plane bending); **^{1}H NMR (400 MHz, $CDCl_3$)**: δ 8.18 (1H, s, H-5'), 7.89 (1H, t, J=1.76 Hz, H-13'), 7.75-7.73 (3H, m, H-15', H-7' and H-11'), 7.45 (2H, t, J=7.52 Hz, H-8' and H-10'), 7.38 (1H, t, J=7.52 Hz, H-16'), 7.36 (1H, d, J=7.52 Hz, H-17'), 7.29 (1H, J=7.40 Hz, t, H-9'), 7.27 (1H, s, H-14), 5.91 (1H, s, H-21), 4.81 (1H, s, H-15), 4.70 (1H, dt, J=9.36 Hz 1.96 Hz, H-12), 4.34 (1H, d, J=11.52 Hz, H_a-19), 3.70 (1H, dd, J=12.80 Hz, 4.64 Hz, H-3), 3.65 (1H, d, J=11.56 Hz, H_b-19), 2.47 (1H, m, H_a-11), 2.41-2.34 (1H, m, H_b-11), 2.07-1.99 (1H, m, H_a-1), 1.90-1.81 (3H, m, H_a-7, H-2), 1.61-1.56 (4H, m, H_b-1, H_a-6, H-9, H_b-6), 1.50 (3H, s, H-18), 1.13 (3H, s, H-17), 1.11 (3H, s, H-20), 1.05 -1.02 (2H, m, H-5, H_b-7); **^{13}C NMR (100 MHz, $CDCl_3$)**: δ 172.56 (C-16), δ 149.30 (C-3'), δ 143.21 (C-14), δ 139.90 (C-6'), δ 138.36 (C-13), δ 134.80 (C-14'), δ 134.40 (C-12'), δ 129.74 (C-13') δ 129.41 (C-8' and C-10'), δ 128.27 (C-16'), δ 128.11 (C-15'), δ 127.37 (C-9'), δ 126.74 (C-5'), δ 126.21 (C-17'), δ 120.66 (C-4'), δ 119.31 (C-7' and C-11'), δ 90.27 (C-21), δ 82.72 (C-8), δ 81.79 (C-3), δ 73.08 (C-12), δ 70.53 (C-15), δ 69.82 (C-19), δ 57.97 (C-9), δ 51.67 (C-5), δ 38.21 (C-4), δ 36.41 (C-7), δ 35.93 (C-10), δ 35.64 (C-1), δ 32.78 (C-11), δ 31.79 (C-17), δ 26.04 (C-18), δ 20.92 (C-2), δ 17.34 (C-6), δ 16.42 (C-20); **HRMS (*m/z*):** Found $[M+H]^+$ = m/z 615.2558, $[M+H+2]^+$ = m/z 617.2558, calculated $[M]^+$ = m/z 614.2548.

3,19-(N-phenyl-3-(3-bromophenyl)-pyrazole) acetal of isoandrographolide (2e)

White solid, yield 80%, m.p. 240.1-242.0°C; **UV (λ_{max})**: 275 nm; **IR (KBr) cm^{-1}:** 3070 (aromatic C-H stretch), 2941 (sp^3 C-H stretch), 1756 (C=O stretch), 1601 (conjugated C=C stretch), 1453 (aromatic C=C stretching), 1203 and 1104 (C-O stretch), 1016 (aromatic C-Br stretch), 878 & 754 (aromatic C-H out of plane bending); **^{1}H NMR (400 MHz, $CDCl_3$)**: δ 8.18 (1H, s, H-5'), 8.04 (1H, t, J=1.60 Hz, H-13'), 7.79 (1H, d, J=7.80 Hz, H-15'), 7.74 (2H, d, J=7.60 Hz, H-7'and H-11'), 7.51 (1H, d, J=7.80 Hz, H-17'), 7.45 (2H, t, J=7.56 Hz, H-8' and H-10'), 7.34-7.28 (3H, m, H-9', H-16', H-14), 5.91 (1H, s, H-21), 4.81 (2H, s, H-15), 4.71 (1H, dt, J=9.28 Hz, 1.88 Hz, H-12), 4.34 (1H, d, J=11.52 Hz, H_a-19), 3.70 (1H, dd, J=12.96 Hz, 4.60 Hz, H-3), 3.65 (1H, d, J=11.64 Hz, H_b-19), 2.48 (1H, m, H_a-11), 2.41-2.34 (1H, m, H_b-11), 2.07-1.99 (1H, m, H_a-1), 1.91-1.83 (3H, m, H_a-7, H-2), 1.60-1.57 (4H, m, H_b-1, H_a-6, H-9, H_b-6), 1.50 (3H, s, H-18), 1.13 (3H, s, H-17), 1.12 (3H, s, H-20), 1.05 -1.02 (2H, m, H-5, H_b-7); **^{13}C NMR (100 MHz, $CDCl_3$)**: δ 172.55 (C-16), δ 149.15 (C-3'), δ 143.18 (C-14), δ 139.86 (C-6'), δ 138.34 (C-13), δ 135.03 (C-12'), δ 131.07 (C-16'), δ 131.01 (C-13'), δ 130.01 (C-15'), δ 129.39 (C-8'), δ 127.34 (C-9'), δ 126.73 (C-5'), δ 126.65 (C-17'), δ 122.59 (C-14'), δ 120.63 (C-4'), δ 119.29 (C-7'), δ 90.23 (C-21), δ 82.70 (C-8), δ 81.77 (C-3), δ 73.06 (C-12), δ 70.52 (C-15), δ 69.81 (C-19), δ 57.93 (C-9), δ 51.64 (C-5), δ 38.17 (C-4), δ 36.37 (C-7), δ 35.91 (C-10), δ 35.61 (C-1), δ 32.76 (C-11), δ 31.77 (C-17), δ 26.02 (C-18), δ 20.96 (C-2), δ 17.31 (C-6), δ 16.39 (C-20); **HRMS (*m/z*):** Found $[M+H]^+$ = m/z 659.2070, $[M+H+2]^+$ = m/z 661.2070, calculated $[M]^+$ = m/z 658.2042.

3,19-(N-phenyl-3-(4-fluorophenyl)-pyrazole) acetal of isoandrographolide (2f):

White solid, yield 86%, m.p. 233.5-236.2°C; **UV (λ_{max})**: 275 nm; **IR (KBr) cm^{-1}:** 3085 (aromatic C-H stretch), 2935 (sp^3 C-H stretch), 1758 (C=O stretch), 1600 (conjugated C=C stretch), 1460 (aromatic C=C stretch), 1222 (aromatic C-F stretch), 1220 and 1100 (C-O stretch), 881 & 760 (aromatic C-H out of plane bending); **^{1}H NMR (400 MHz, $CDCl_3$)**: δ 8.18 (1H, s, H-5'), 7.83-7.79 (2H, m, H-13' and H-17'), 7.74 (2H, d, J=7.76 Hz, H-7' and H-11') 7.44 (2H, t, J=7.64 Hz, H-8' and H-10'), 7.28 (2H, t, J=7.60 Hz, H-9', H-14), 7.14 (2H, t, J=8.72 Hz, H-14' and H-16'), 5.88 (1H, s, H-21), 4.81 (2H, bs, H-15), 4.71 (1H, t, J=7.36 Hz, H-12), 4.33 (1H, d, J=11.48 Hz, H_a-19), 3.68 (1H, dd, J=12.80 Hz, 4.64 Hz, H-3), 3.65 (1H, d, J=11.72 Hz, H_b-19), 2.47 (1H, m, H_a-11), 2.41-2.31 (1H, m, H_b-11), 2.07-1.99 (1H, m, H_a-1), 1.88-1.71 (3H, m, H_a-7, H-2), 1.59-1.56 (4H, m, H_b-1, H_a-6, H-9, H_b-6), 1.50 (3H, s, H-18), 1.13 (3H, s, H-17), 1.11 (3H, s, H-20), 1.05 -1.02 (2H, m, H-5, H_b-7); **^{13}C NMR (100 MHz,**

$CDCl_3$): δ 170.60 (C-16), δ 162.86 (d, C-15', J=245.59 Hz), δ 149.90 (C-3'), δ 143.27 (C-14), δ 139.95 (C-6'), δ 138.35 (C-13), δ 130.00 (d, C-13' and C-17', J=8.04 Hz), δ 129.39 (C-8' and C-10'), δ 129.17 (d, C-12', J=2.97 Hz), δ 127.23 (C-9'), δ 126.62 (C-5'), δ 120.36 (C-4'), δ 119.26 (C-7' and C-11'), δ 115.45 (d, C-14' and C-16', J=21.41 Hz), δ 90.34 (C-21), δ 82.73 (C-8), δ 81.67 (C-3), δ 73.07 (C-12), δ 70.57 (C-15), δ 69.80 (C-19), δ 57.93 (C-9), δ 51.69 (C-5), δ 38.16 (C-4), δ 36.41 (C-7), δ 35.95 (C-10), δ 35.63 (C-1), δ 32.75 (C-11), δ 31.78 (C-17), δ 26.03 (C-18), δ 20.96 (C-2), δ 17.34 (C-6), δ 16.44 (C-20); **HRMS (*m/z*):** Found $[M+H]^+$ = m/z 599.2859, calculated $[M]^+$ = m/z 598.2843.

3,19-(N-phenyl-3-(4-chlorophenyl)-pyrazole) acetal of isoandrographolide (2g):

White solid, yield 83%, m.p. 216.0-217.7°C; **UV (λ_{max})**: 280 nm; **IR (KBr) cm^{-1}:** 3089 (aromatic C-H stretch), 2925 (sp^3 C-H stretch), 1758 (C=O stretch), 1600 (conjugated C=C stretch), 1450 (aromatic C=C stretch), 1220 and 1101 (C-O stretch), 1070 (aromatic C-Cl stretch), 878 & 761 (aromatic C-H out of plane bending); **^{1}H NMR (400 MHz, $CDCl_3$)**: δ 8.18 (1H, s, H-5'), 7.78 (2H, d, J=8.48 Hz, H-13' and H-17'), 7.74 (2H, d, J=7.68 Hz, H-7' and H-11') 7.44 (2H, t, J=7.68 Hz, H-8' and H-10'), 7.43 (2H, d, J=8.48 Hz, H-14' and H-16'), 7.29 (2H, t, J=7.52 Hz, H-9', H-14), 5.88 (1H, s, H-21), 4.81 (2H, bs, H-15), 4.71 (1H, dt, J=9.26 Hz, 1.88 Hz, H-12), 4.33 (1H, d, J=11.48 Hz, H_a-19), 3.68 (1H, dd, J=12.80 Hz, 4.68 Hz, H-3), 3.64 (1H, d, J=11.76 Hz, H_b-19), 2.47 (1H, m, H_a-11), 2.41-2.31 (1H, m, H_b-11), 2.07-2.00 (1H, m, H_a-1), 1.89-1.72 (3H, m, H_a-7, H-2), 1.60-1.52 (4H, m, H_b-1, H_a-6, H-9, H_b-6), 1.49 (3H, s, H-18), 1.13 (3H, s, H-17), 1.11 (3H, s, H-20), 1.05 -1.02 (2H, m, H-5, H_b-7). **^{13}C NMR (100 MHz, $CDCl_3$)**: δ 172.60 (C-16), δ 149.62 (C-3'), δ 143.28 (C-14), δ 139.91 (C-6'), δ 138.35 (C-13), δ 134.10 (C-15'), δ 131.54 (C-12'), δ 129.50 (C-8' and C-10'), δ 129.41 (C-14' and C-16'), δ 128.68 (C-13' and C-17'), δ 127.36 (C-9'), δ 126.70 (C-5'), δ 120.52 (C-4'), δ 119.30 (C-7' and C-11'), δ 90.29 (C-21), δ 82.73 (C-8), δ 81.68 (C-3), δ 73.07 (C-12), δ 70.57 (C-15), δ 69.79 (C-19), δ 57.93 (C-9), δ 51.69 (C-5), δ 38.15 (C-4), δ 36.42 (C-7), δ 35.95 (C-10), δ 35.63 (C-1), δ 32.75 (C-11), δ 31.78 (C-17), δ 26.03 (C-18), δ 20.96 (C-2), δ 17.34 (C-6), δ 16.44 (C-20); **HRMS (*m/z*):** Found $[M+H]^+$ = m/z 615.2569, $[M+H+2]^+$ = m/z 617.2569, calculated $[M]^+$ = m/z 614.2548.

3,19-(N-phenyl-3-(4-bromophenyl)-pyrazole) acetal of isoandrographolide (2h):

White solid, yield 88%, m.p. 204.2-206.2°C; **UV (λ_{max})**: 280 nm; **IR (KBr) cm^{-1}:** 3077 (aromatic C-H stretch), 2934 (sp^3 C-H stretch), 1758 (C=O stretch), 1600 (conjugated C=C stretch), 1450 (aromatic C=C stretch), 1221 and 1100 (C-O stretch), 1020 (aromatic C-Br stretch), 875 & 762 (aromatic C-H out of plane bending); **^{1}H NMR (400 MHz, $CDCl_3$)**: δ 8.18 (1H, s, H-5'), 7.74-7.71 (4H, m, H-13', H-17', H-7' and H-11'), 7.58 (2H, d, J=8.48 Hz, H-14' and H-16'), 7.44 (2H, t, J=7.60 Hz, H-8' and H-10'), 7.29 (1H, t, J=7.76 Hz, H-9'), 7.28 (1H, s, H-14), 5.88 (1H, s, H-21), 4.81 (2H, bs, H-15), 4.71 (1H, t, J=7.4 Hz, H-12), 4.32 (1H, d, J=11.48 Hz, H_a-19), 3.68 (1H, dd, J=12.80 Hz, 4.56 Hz, H-3), 3.64 (1H, d, J=11.60 Hz H_b-19), 2.47 (1H, m, H_a-11), 2.41-2.31 (1H, m, H_b-11), 2.07-2.00 (1H, m, H_a-1), 1.89-1.86 (3H, m, H_a-7, H-2), 1.76-1.71 (3H, m, H_b-1, H_a-6, H-9), 1.59-1.56 (1H, m, H_b-6), 1.49 (3H, s, H-18), 1.13 (3H, s, H-17), 1.11 (3H, s, H-20), 1.05-1.02 (2H, m, H-5, H_b-7). **^{13}C NMR (100 MHz, $CDCl_3$)**: δ 172.56 (C-16), δ 149.62 (C-3'), δ 143.25 (C-13), δ 139.92 (C-6'), δ 138.36 (C-13), δ 132.02 (C-12'), δ 131.61 (C-14' and C-16'), δ 129.80 (C-8' and C-10'), δ 129.40 (C-13' and C-17'), δ 127.38 (C-9'), δ 126.70 (C-5'), δ 122.36 (C-15'), δ 120.54 (C-4'), δ 119.30 (C-7' and C-11'), δ 90.29 (C-21), δ 82.72 (C-8), δ 81.68 (C-3), δ 73.07 (C-12), δ 70.54 (C-15), δ 69.78 (C-19), δ 57.96 (C-9), δ 51.69 (C-5), δ 38.17 (C-4), δ 36.42 (C-7), δ 35.94 (C-10), δ 35.65 (C-1), δ 32.75 (C-11), δ 31.78 (C-17), δ 26.03 (C-18), δ 20.97 (C-2), δ 17.34 (C-6), δ 16.44 (C-20); **HRMS (*m/z*):** Found $[M+H]^+$ = m/z 659.2056, $[M+H+2]^+$ = m/z 661.2056, calculated $[M]^+$ = m/z 658.2042.

3,19-(N-phenyl-3-(4-methylphenyl)-pyrazole) acetal of isoandrographolide (2i)

White solid, yield 90%, m.p. 151.1-152.3°C; **UV (λ_{max})**: 275 nm; **IR (KBr) cm^{-1}:** 3072 (aromatic C-H stretch), 2932 (sp^3 C-H stretch), 1758 (C=O stretch), 1600 (conjugated C=C stretch), 1461 (aromatic C=C stretch), 1222 and 1098 (C-O stretch), 870 & 756 (aromatic C-H out of plane bending); **^{1}H NMR (400 MHz, $CDCl_3$)**: δ 8.19 (1H, s, H-5'), 7.75 (2H, d, J=7.72 Hz, H-7'and H-11'), 7.72 (2H, d, J=8.04 Hz, H-13' and H-17'), 7.43 (1H, t, J=7.64 Hz, H-9'), 7.28-7.26 (5H, m, H-8', H-10', H-14', H-16' and H-14), 5.90 (1H, s, H-21), 4.81 (2H, bs, H-15), 4.70 (1H, dt, J=7.48 Hz, J=2.12 Hz, H-12), 4.32 (1H, d, J=11.48 Hz, H_a-19), 3.69 (1H, dd, J=12.68 Hz, J=4.6 Hz, H-3), 3.64 (1H, d, J=11.64 Hz, H_b-19), 2.47 (1H, m, H_a-11), 2.41 (3H, s, H-18'), 2.24-2.20 (1H, m, H_b-11), 2.07-1.99 (1H, m, H_a-1), 1.86 (3H, m, H_a-7, H-2), 1.78-1.73 (3H, m, H_b-1, H_a-6, H-9), 1.58-1.56 (1H, m, H_b-6), 1.51 (3H, s, H-18), 1.13 (3H, s, H-17), 1.10 (3H, s, H-20), 1.05 -1.02 (2H, m, H-5, H_b-7); **^{13}C NMR (100 MHz, $CDCl_3$)**: δ 172.58 (C-

16), δ 150.85 (C-3’), δ 143.22 (C-14), δ 140.05 (C-6’), δ 138.33 (C-12), δ 137.93 (C-15’), δ 130.11 (C-12’), δ 129.31 (C-14’ and C-16’), δ 129.20 (C-8’ and C-10’), δ 128.08 (C-13’ and C-17’), δ 126.99 (C-9’), δ 126.42 (C-5’), δ 120.32 (C-4’), δ 119.24 (C-7’ and C-11’), δ 90.43 (C-21), δ 82.71 (C-8), δ 81.66 (C-3), δ 73.04 (C-12), δ 70.54 (C-15), δ 69.78 (C-19), δ 57.91 (C-9), δ 51.66 (C-1), δ 38.17 (C-4), δ 36.39 (C-7), δ 35.93 (C-10), δ 35.61 (C-1), δ 32.73 (C-11), δ 31.76 (C-17), δ 26.02 (C-18), δ 21.32 (C-18’), δ 20.95 (C-2), δ 17.31 (C-6), δ 16.39 (C-20); **HRMS (*m/z*):** Found $[M+H]^+$ = m/z 595.3120, calculated $[M]^+$ = m/z 594.3094.

3,19-(N-phenyl-3-(4-methoxyphenyl)-pyrazole) acetal of isoandrographolide (2j)

White solid, yield 88%, m.p. 114.3-115.9°C; **UV (λ_{max})**: 280 nm; **IR (KBr) cm^{-1}:** 3078 (aromatic C-H stretch), 2936 (sp^3 C-H stretch), 1758 (C=O stretch), 1600 (conjugated C=C stretch), 1461 (aromatic C=C stretch), 1251, 1220 and 1099 (C-O stretch), 887 & 758 (aromatic C-H out of plane bending); **^{1}H NMR (400 MHz, $CDCl_3$)**: δ 8.17 (1H, s, H-5’), 7.75 (2H, d, J=7.64 Hz, H-7’ and H-11’), 7.72 (2H, d, J=8.84 Hz, H-13’ and H-17’), 7.43 (2H, t, J=7.56 Hz, H-8’ and H-10’), 7.28-7.27 (2H, m, H-9’, H-14), 6.99 (2H, d, J=8.84 Hz, H-14’ and H-16’), 5.89 (1H, s, H-21), 4.81 (1H, s, H-15), 4.70 (1H, dt, J=9.32 Hz, J=2.08 Hz, H-12), 4.33 (1H, d, J=11.48 Hz, H_a-19), 3.87 (3H, s, H-18’), 3.68 (1H, dd, J=12.72 Hz, 4.6 Hz, H-3), 3.64 (1H, d, J=11.8 Hz H_b-19), 2.47 (1H, m, H_a-11), 2.39-2.20 (1H, m, H_b-11), 2.07-1.99 (1H, m, H_a-1), 1.87 (3H, m, H_a-7, H-2), 1.77-1.73 (3H, m, H_b-1, H_a-6, H-9), 1.64-1.56 (1H, m, H_b-6), 1.51 (3H, s, H-18), 1.12 (3H, s, H-17), 1.08 (3H, s, H-20), 1.05 -1.01 (2H, m, H-5, H_b-7); **^{13}C NMR (100 MHz, $CDCl_3$)**: δ 172.59 (C-16), δ 159.67 (C-15’), δ 150.65 (C-3’), δ 143.24 (C-14), δ 140.08 (C-6’), δ 138.36 (C-13), δ 129.48 (C-8’ and C-10’), δ 129.33 (C-13’ and C-17’), δ 126.98 (C-9’), δ 126.39 (C-5’), δ 125.65 (C-12’), δ 120.17 (C-4’), δ 119.21 (C-7’ and C-11’), δ 113.92 (C-14’ and C-16’), δ 90.49 (C-21), δ 82.74 (C-8), δ 81.67 (C-3), δ 73.07 (C-12), δ 70.55 (C-15), δ 69.80 (C-19), δ 57.96 (C-9), δ 55.35 (C-18’), δ 51.69 (C-5), δ 38.21 (C-4), δ 36.42 (C-7), δ 35.95 (C-10), δ 35.64 (C-1), δ 32.76 (C-11), δ 31.75 (C-17), δ 26.04 (C-18), δ 20.98 (C-2), δ 17.34 (C-6), δ 16.43 (C-20); **HRMS (*m/z*):** Found $[M+H]^+$ = m/z 611.3059, calculated $[M]^+$ = m/z 610.2041.

Spectra of all the compounds

3,19-(N-phenyl-3-(phenyl)-pyrazole) acetal of andrographolide (1a):

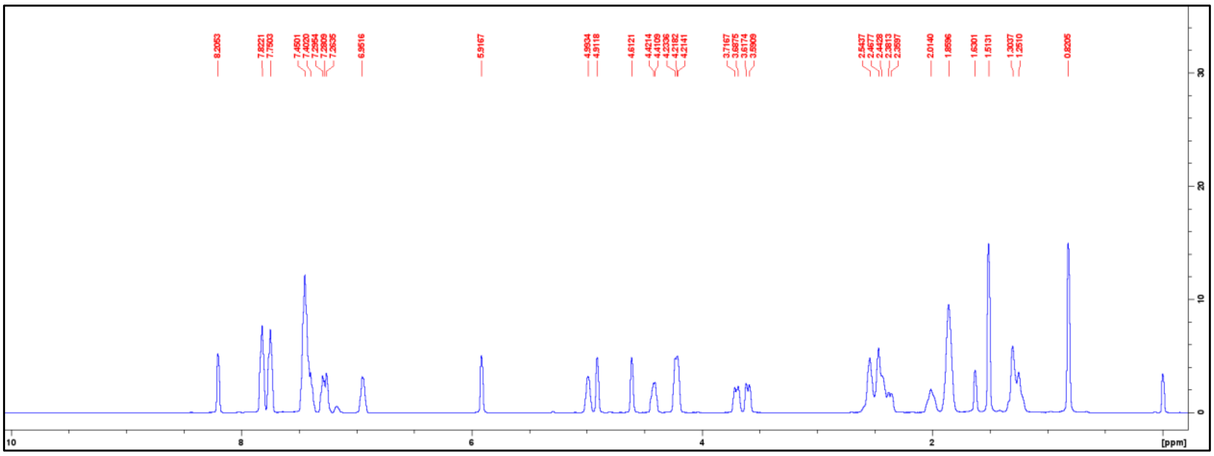

Figure 1SI: ^{1}H NMR spectrum of **1a**

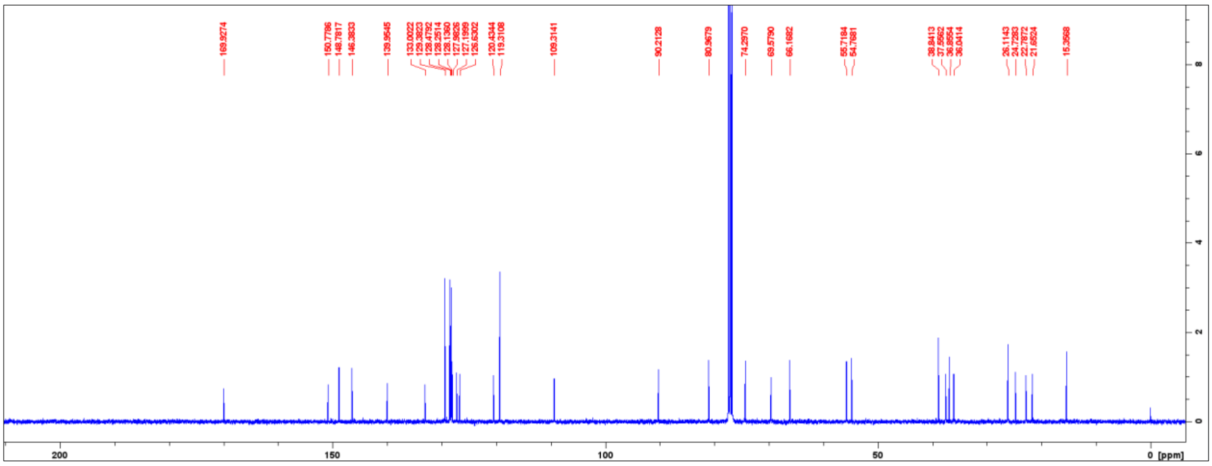

Figure 2SI: ^{13}C NMR spectrum of **1a**

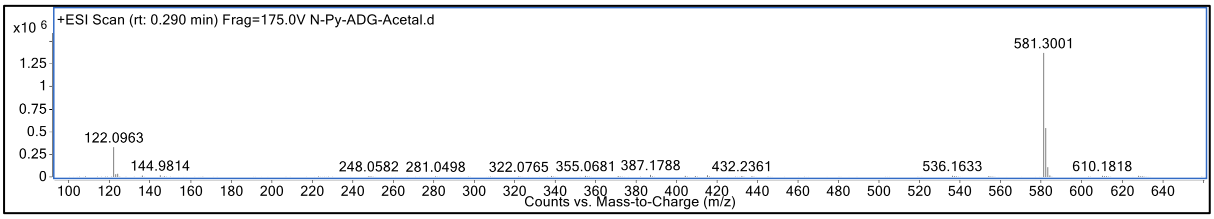

Figure 3SI: HRMS of **1a**

3,19-(N-phenyl-3-(3-nitrophenyl)-pyrazole) acetal of andrographolide (1b):

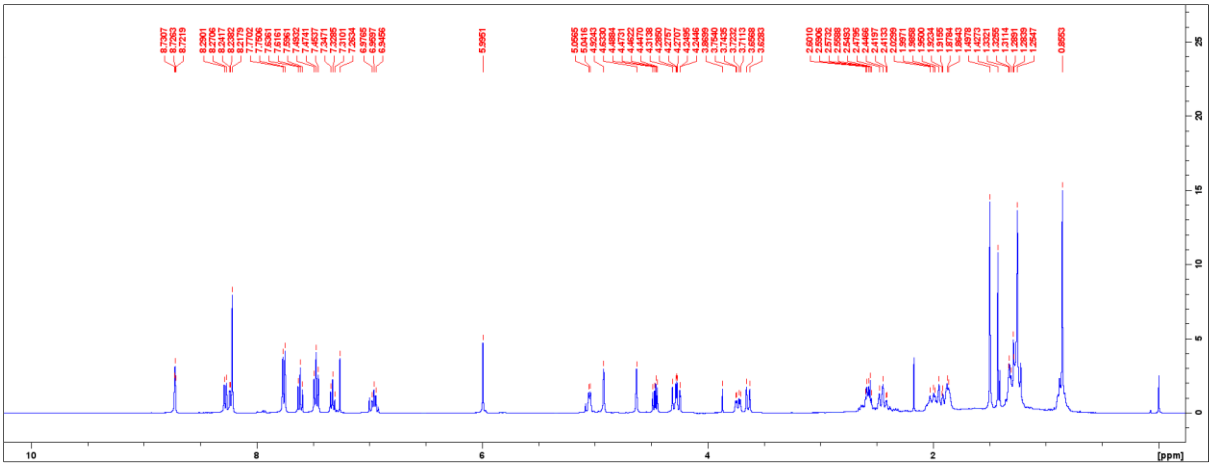

Figure 4SI: ^{1}H NMR spectrum of **1b**

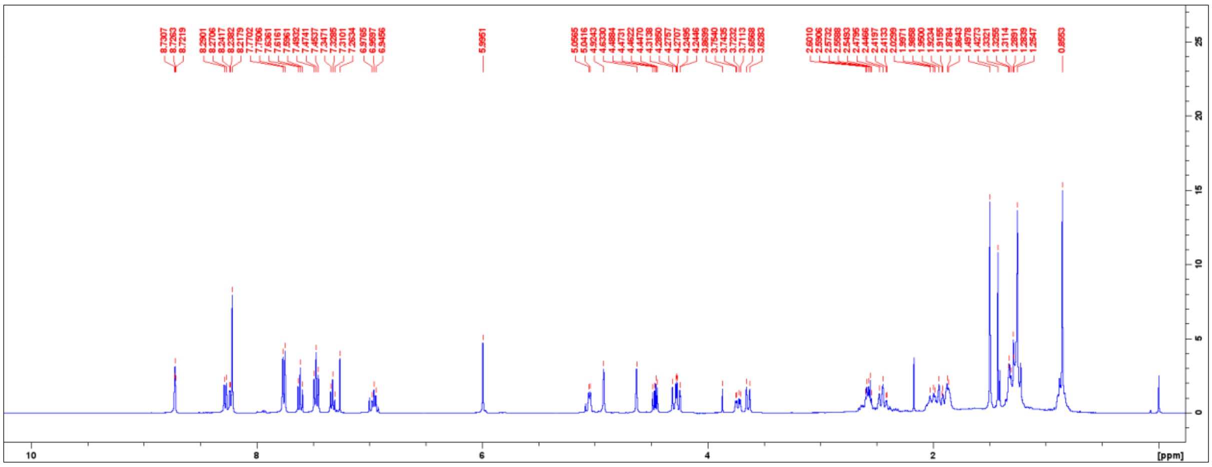

Figure 5SI: ^{13}C NMR spectrum of **1b**

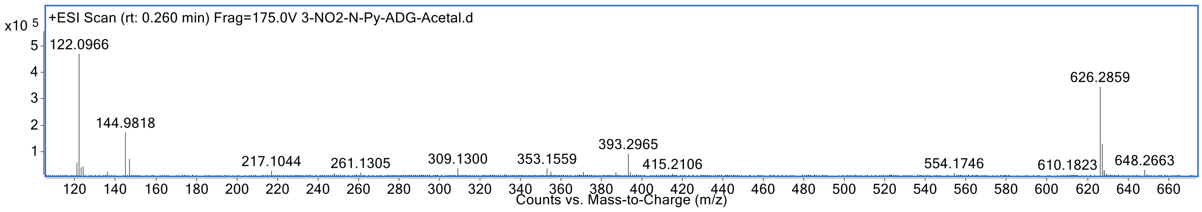

Figure 6SI: HRMS of **1b**

3,19-(N-phenyl-3-(3-fluorophenyl)-pyrazole) acetal of andrographolide (1c):

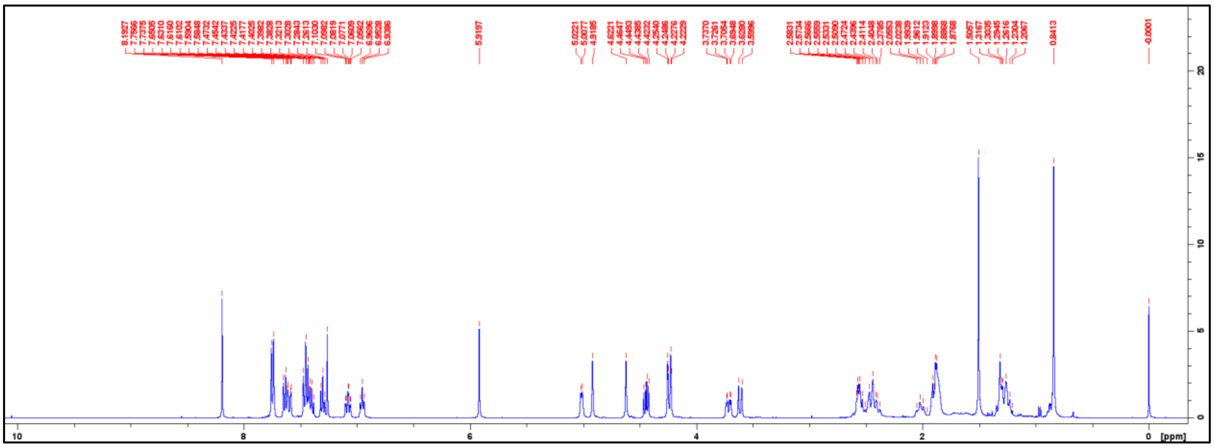

Figure 7SI: ^{1}H NMR spectrum of **1c**

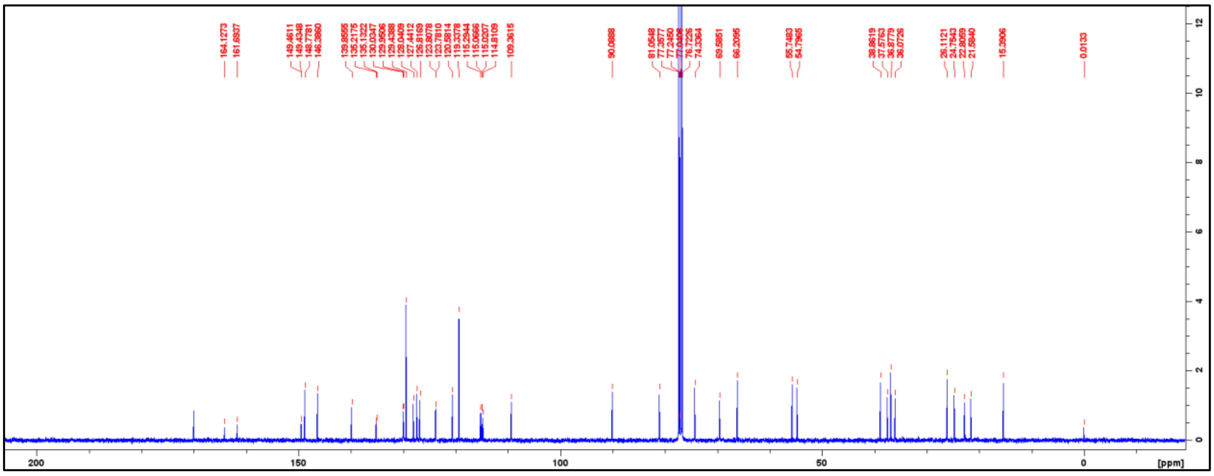

Figure 8SI: ^{13}C NMR spectrum of **1c**

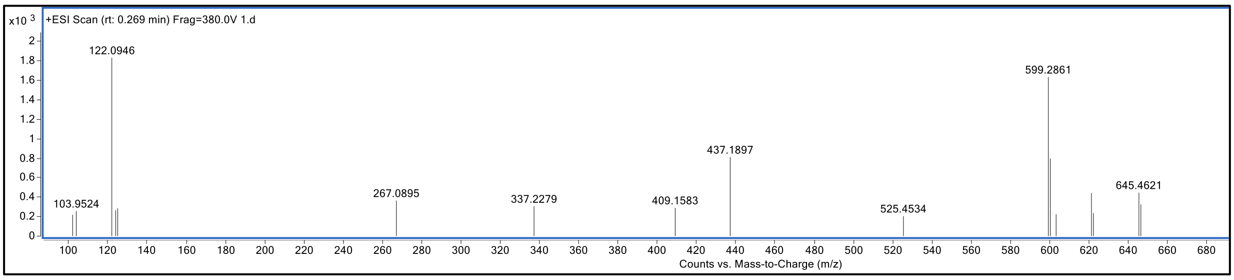

Figure 9SI: HRMS of **1c**

3,19-(N-phenyl-3-(3-chlorophenyl)-pyrazole) acetal of andrographolide (1d)

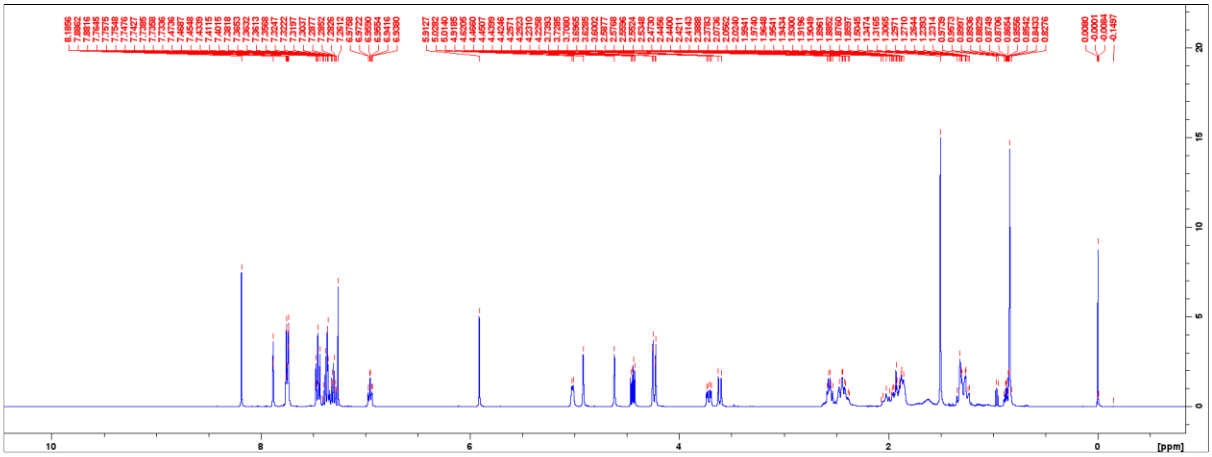

Figure 10SI: ^{1}H NMR spectrum of **1d**

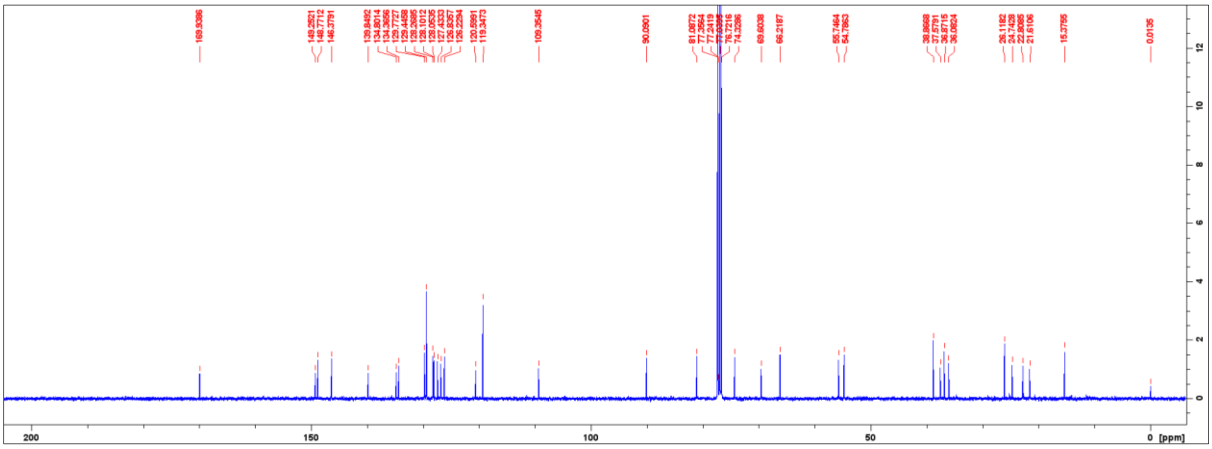

Figure 11SI: ^{13}C NMR spectrum of **1d**

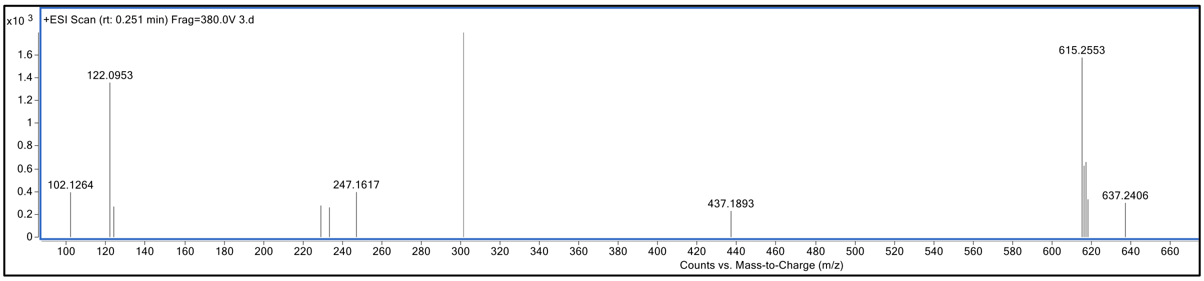

Figure 12SI: HRMS of **1d**

3,19-(N-phenyl-3-(3-bromophenyl)-pyrazole) acetal of andrographolide (1e):

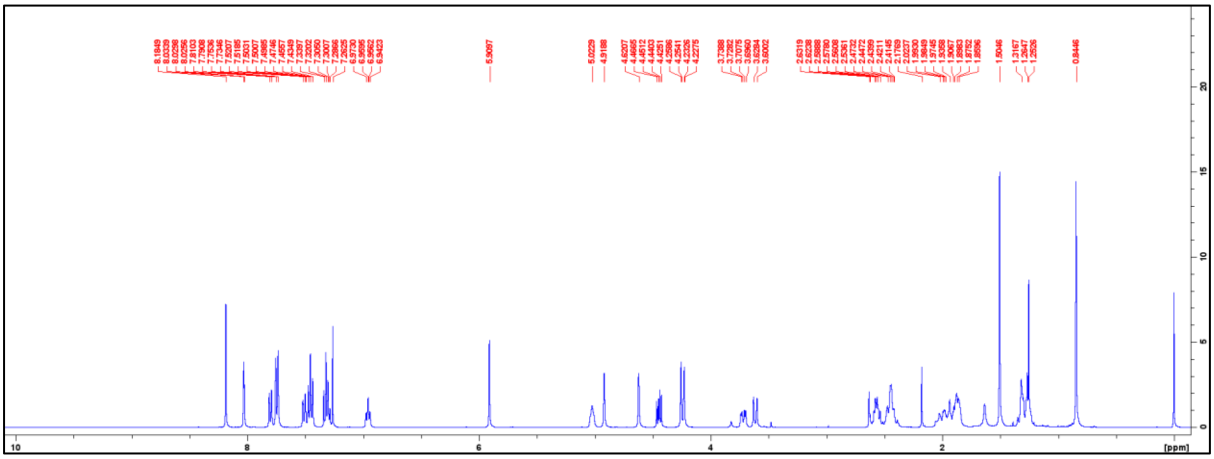

Figure 13SI: ^{1}H NMR spectrum of **1e**

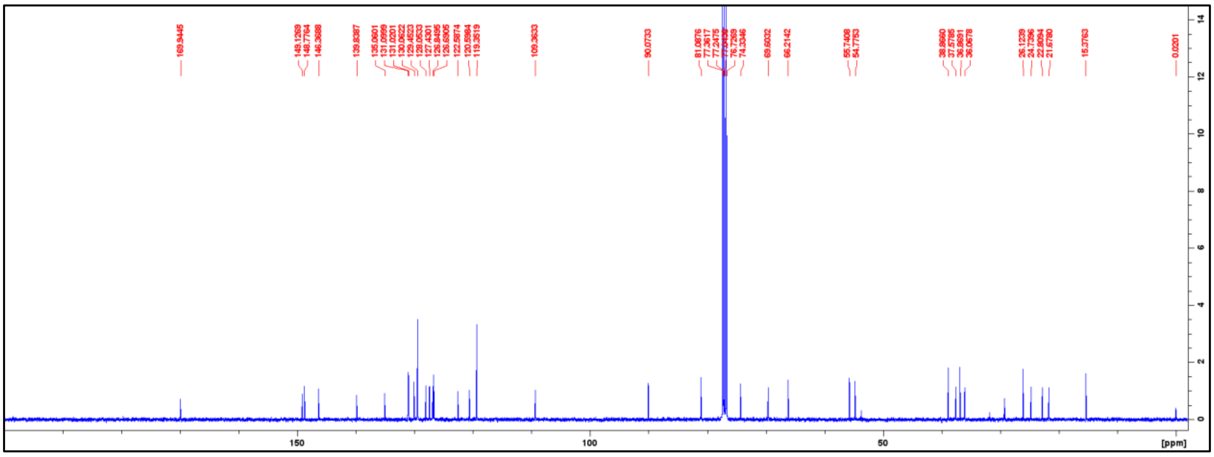

Figure 14SI: ^{13}C NMR spectrum of **1e**

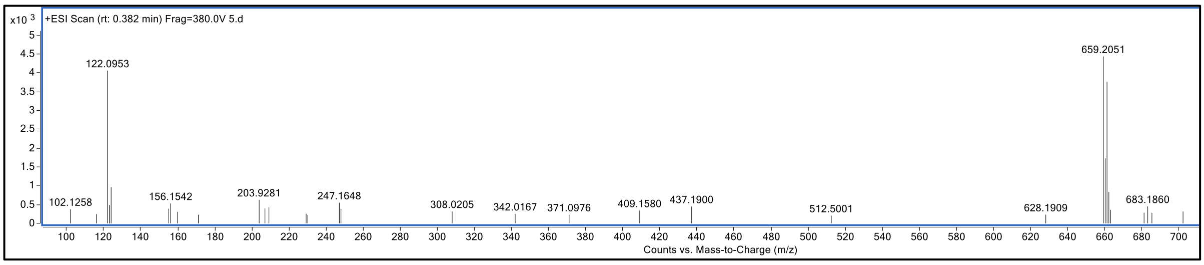

Figure 15SI: HRMS of **1e**

3,19-(N-phenyl-3-(4-fluorophenyl)-pyrazole) acetal of andrographolide (1f):

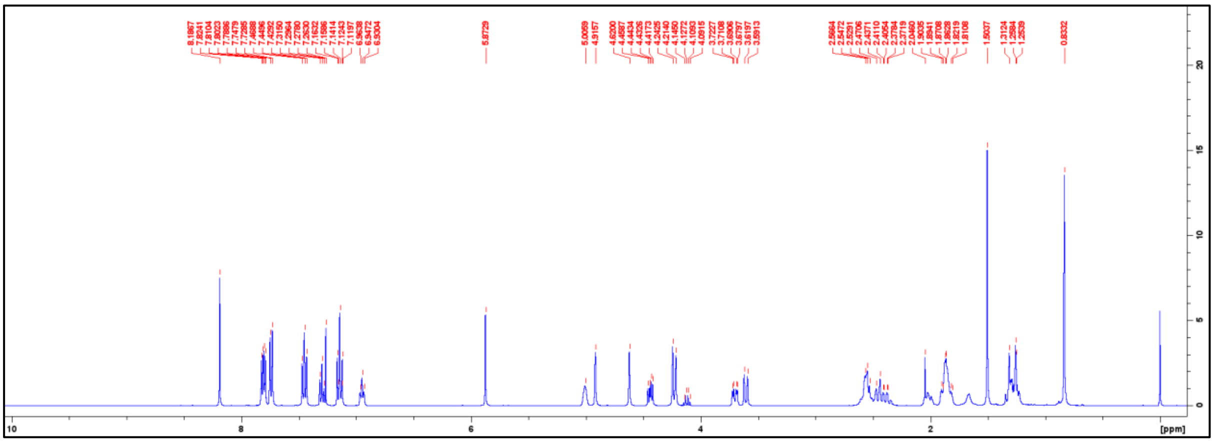

Figure 16SI: ^{1}H NMR spectrum of **1f**

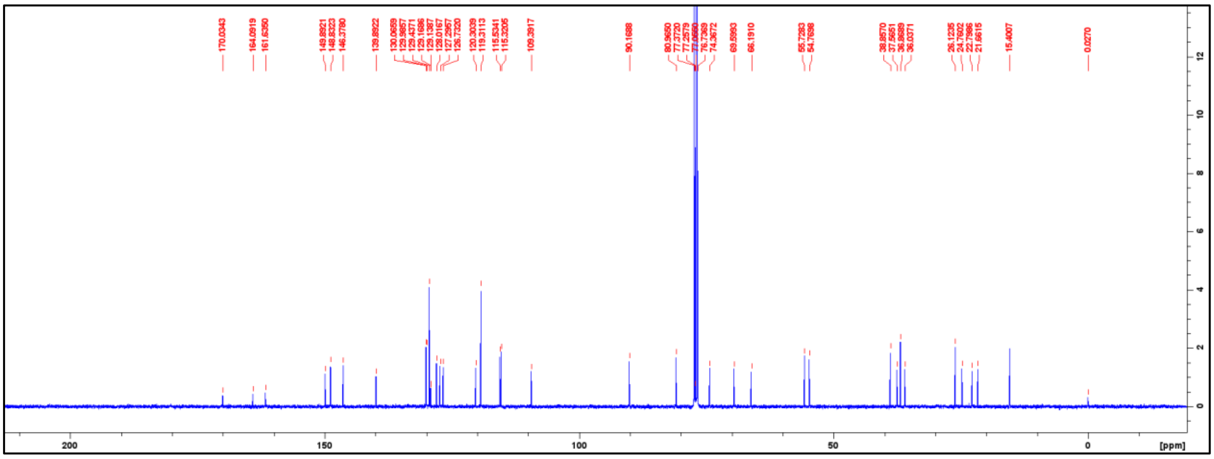

Figure 17SI: ^{13}C NMR spectrum of **1f**

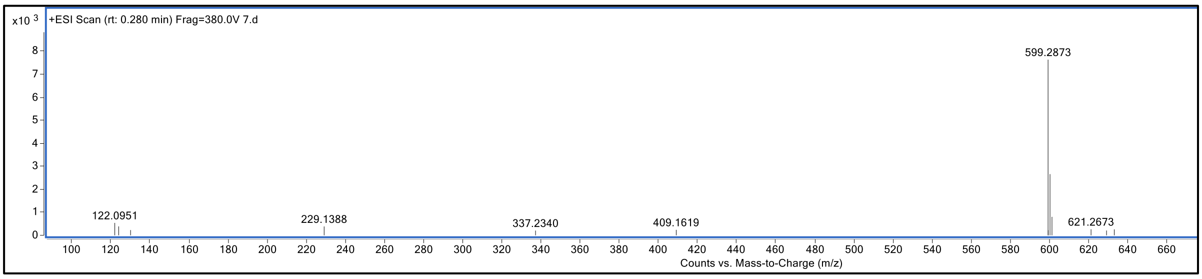

Figure 18SI: HRMS of **1f**

3,19-(N-phenyl-3-(4-chlorophenyl)-pyrazole) acetal of andrographolide (1g):

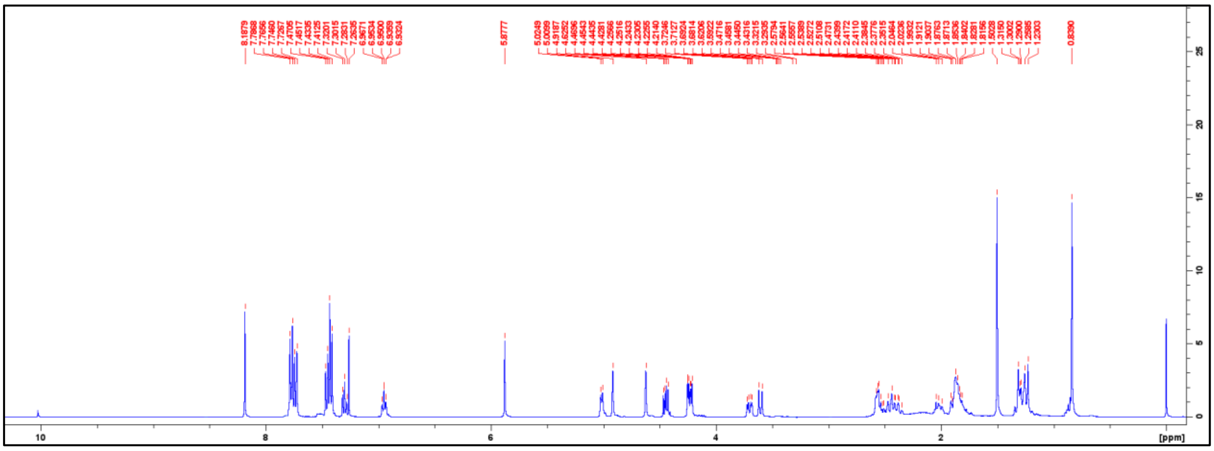

Figure 19SI: ^{1}H NMR spectrum of **1g**

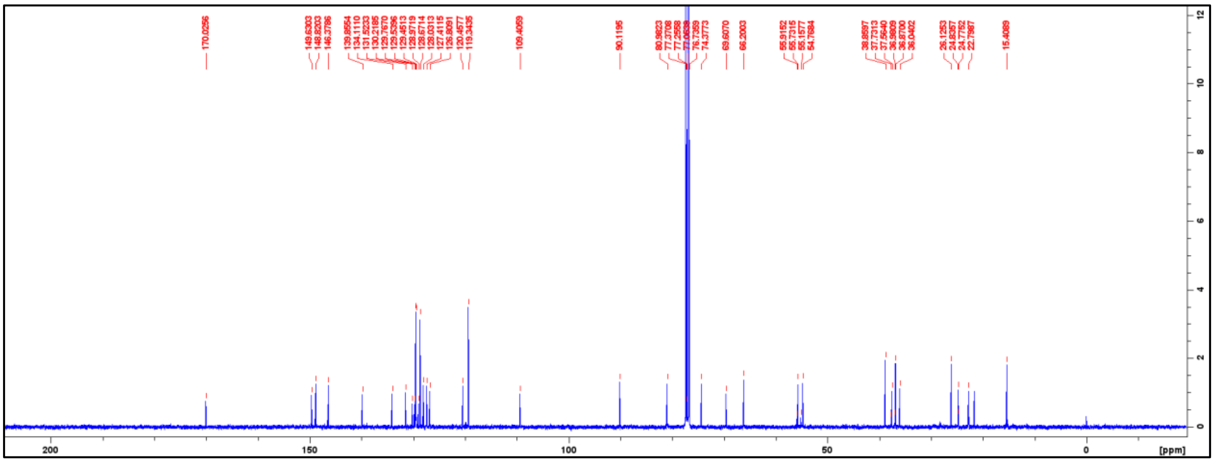

Figure 20SI: ^{13}C NMR spectrum of **1g**

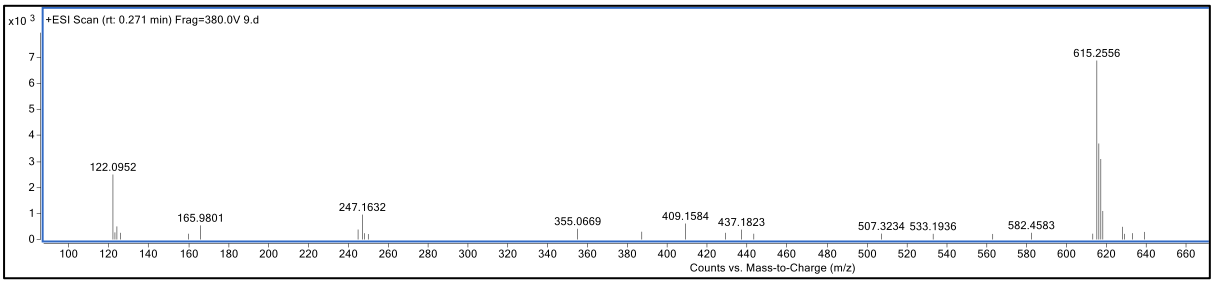

Figure 21SI: HRMS of **1g**

3,19-(N-phenyl-3-(4-bromophenyl)-pyrazole) acetal of andrographolide (1h):

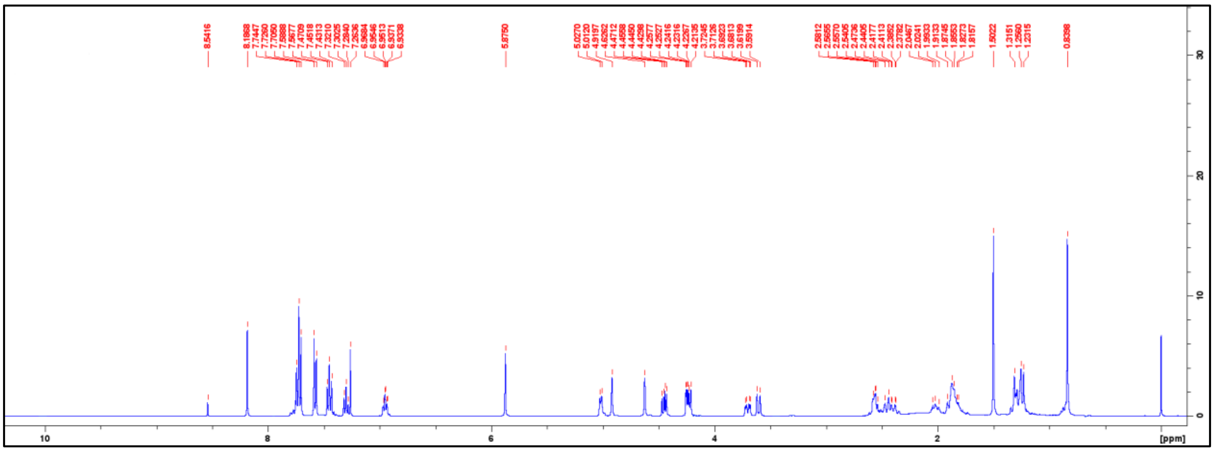

Figure 22SI: ^{1}H NMR spectrum of **1h**

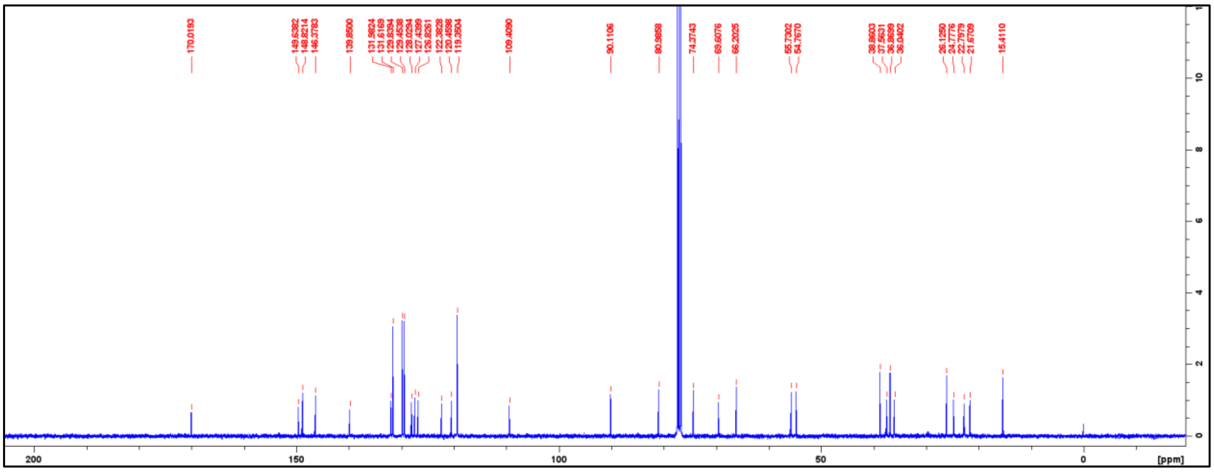

Figure 23SI: ^{13}C NMR spectrum of **1h**

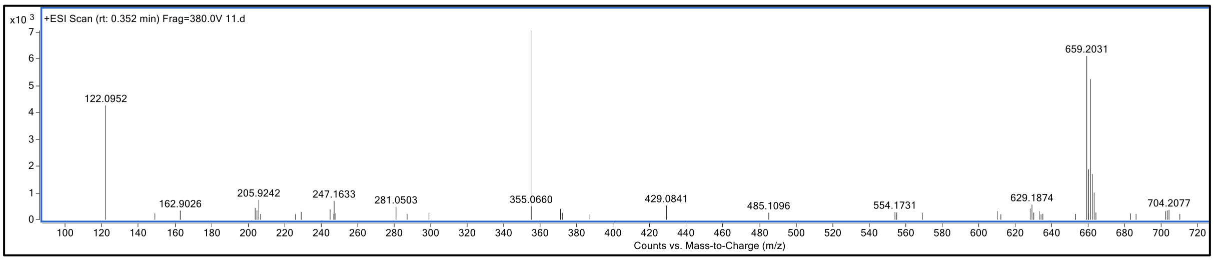

Figure 24SI: HRMS of **1h**

3,19-(N-phenyl-3-(4-methylphenyl)-pyrazole) acetal of andrographolide (1i):

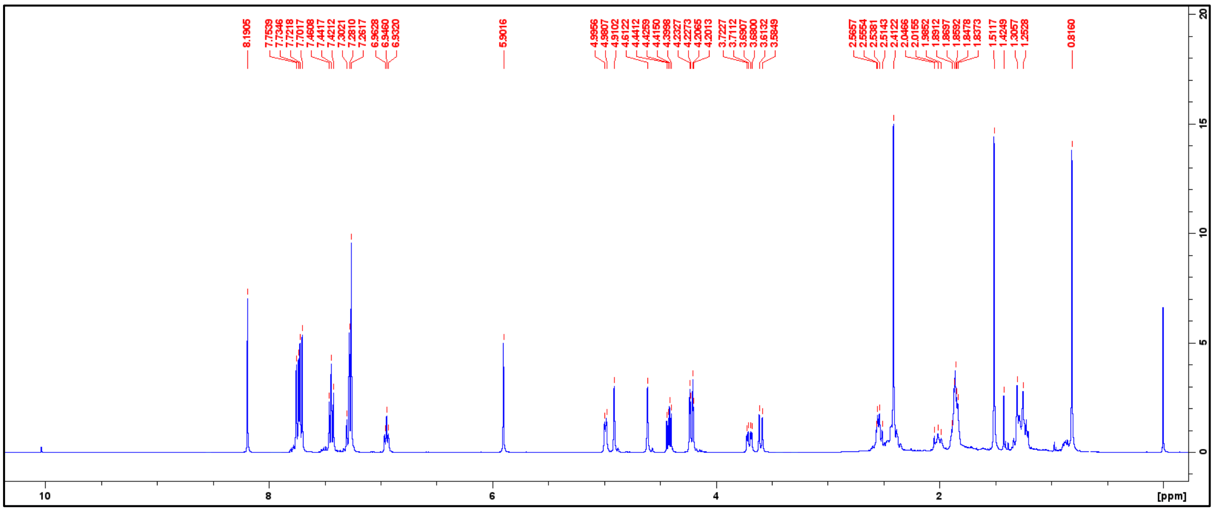

Figure 25SI: ^{1}H NMR spectrum of **1i**

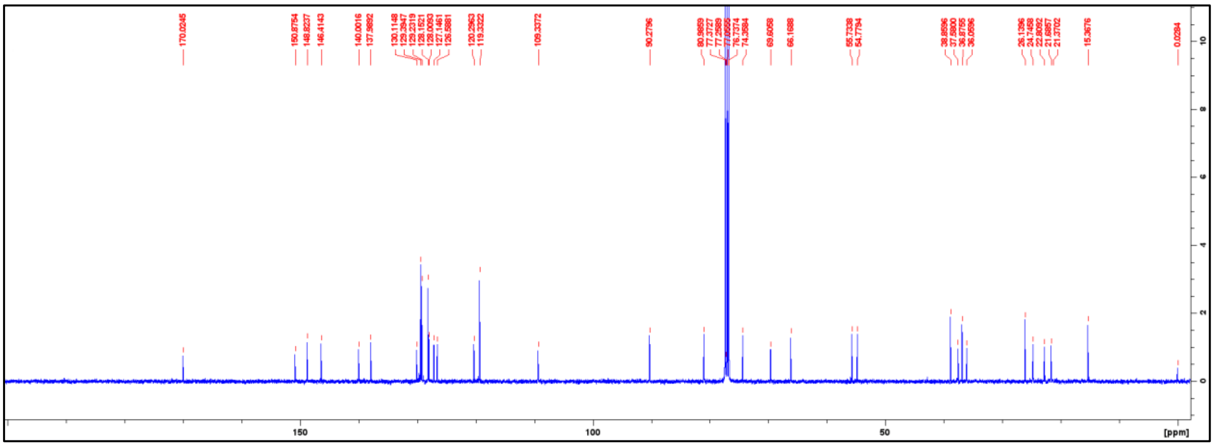

Figure 26SI: ^{13}C NMR spectrum of **1i**

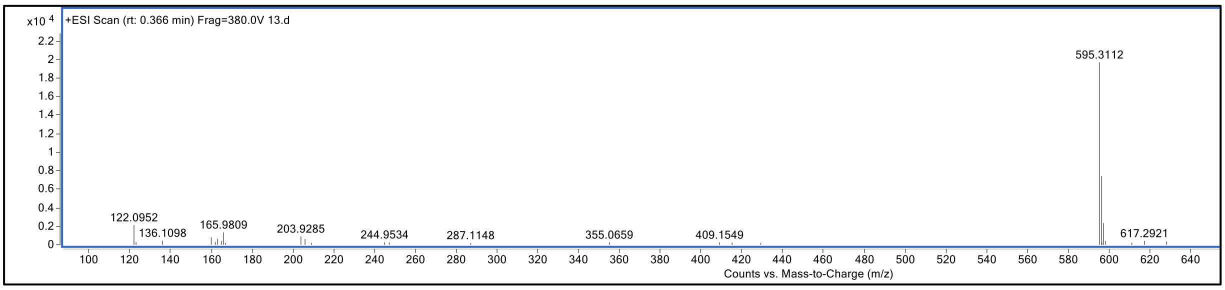

Figure 27SI: HRMS of **1i**

3,19-(N-phenyl-3-(4-methoxyphenyl)-pyrazole) acetal of andrographolide (1j):

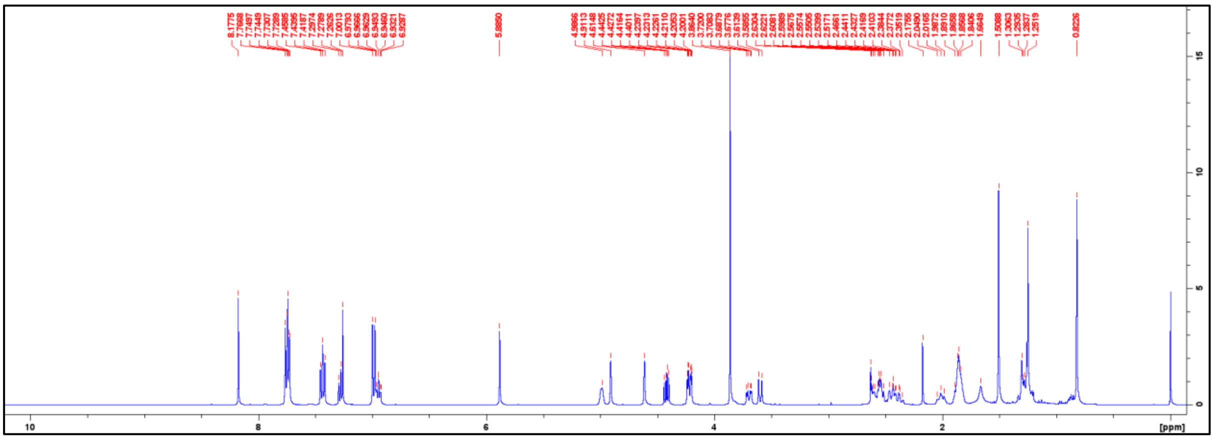

Figure 28SI: ^{1}H NMR spectrum of **1j**

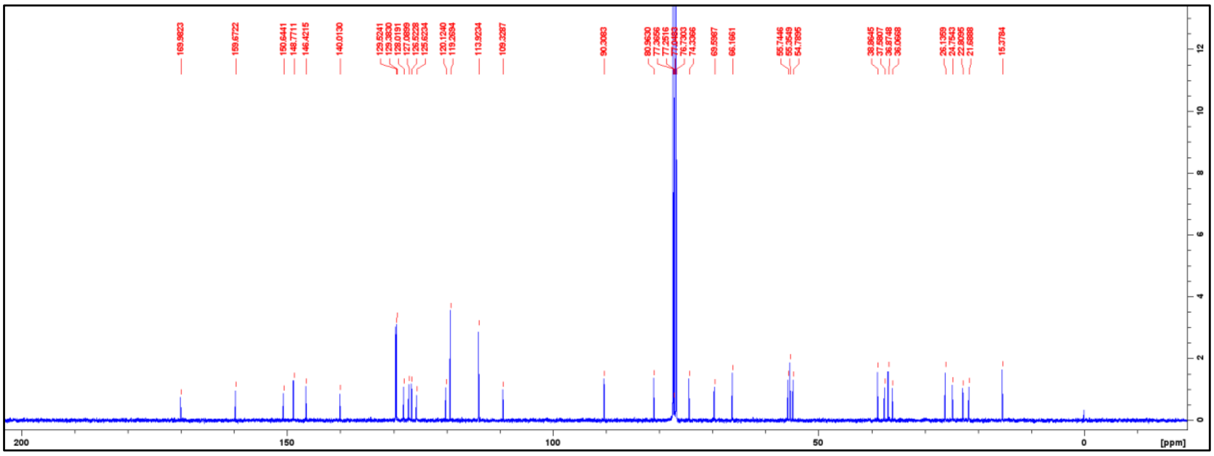

Figure 29SI: ^{13}C NMR spectrum of **1j**

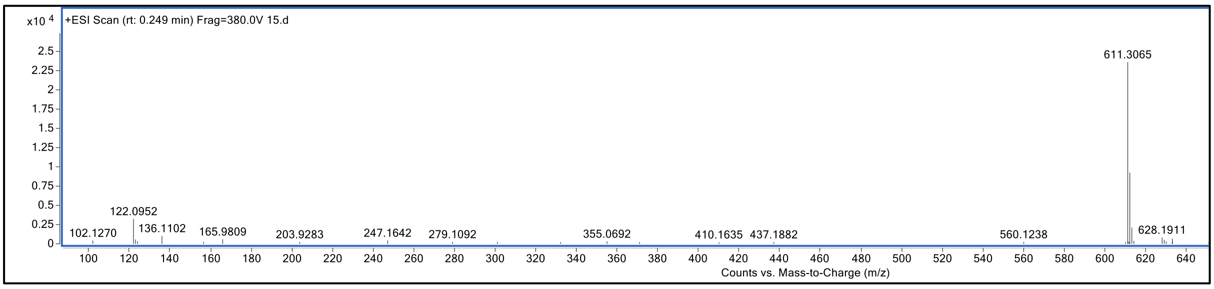

Figure 30SI: HRMS of **1j**

3,19-(N-phenyl-3-(phenyl)-pyrazole) acetal of isoandrographolide (2a):

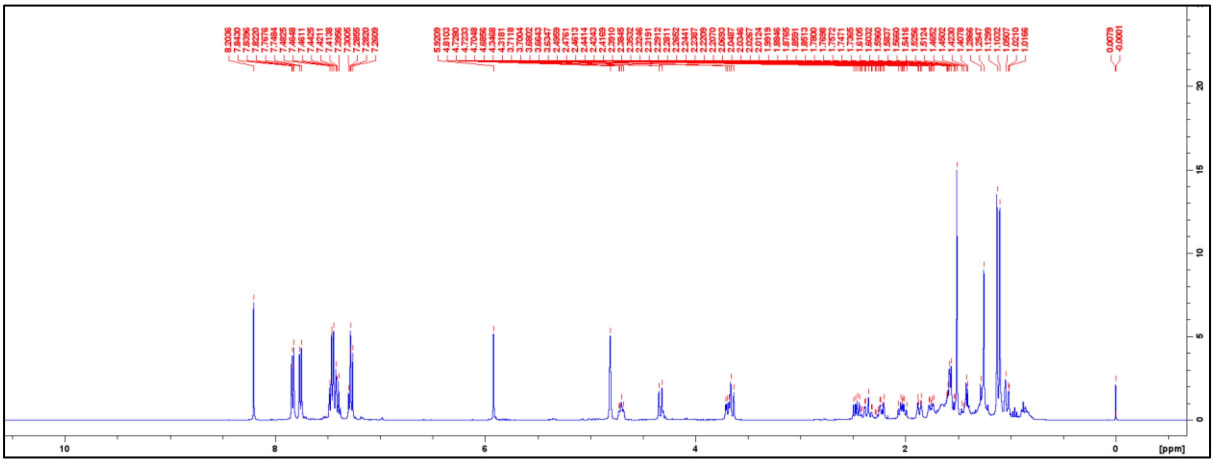

Figure 31SI: ^{1H} NMR spectrum of **2a**

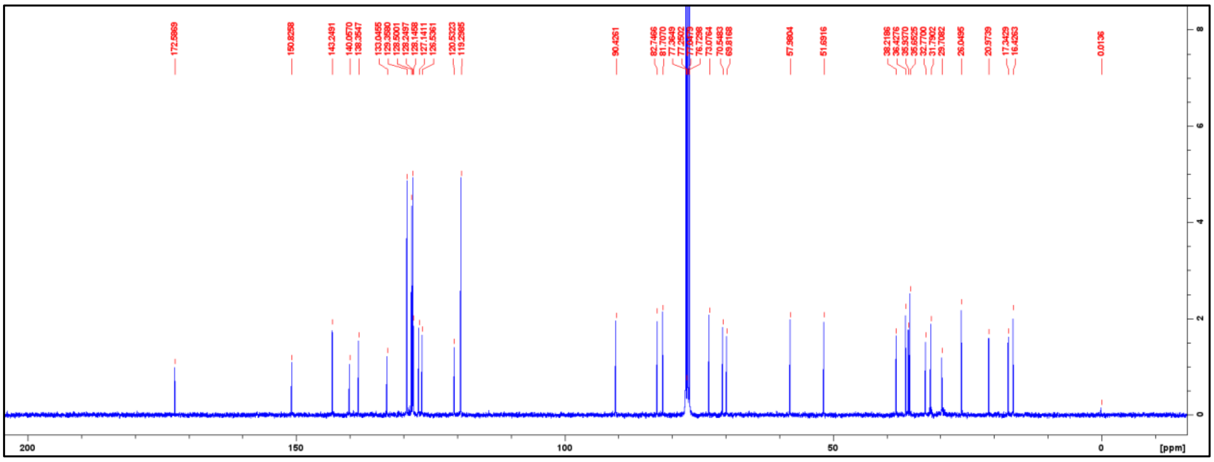

Figure 32SI: ^{13}C NMR spectrum of **2a**

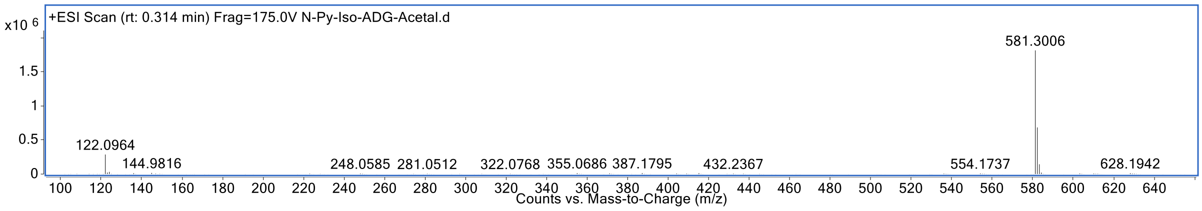

Figure 33SI: HRMS of **2a**

3,19-(N-phenyl-3-(3-nitrophenyl)-pyrazole) acetal of isoandrographolide (2b):

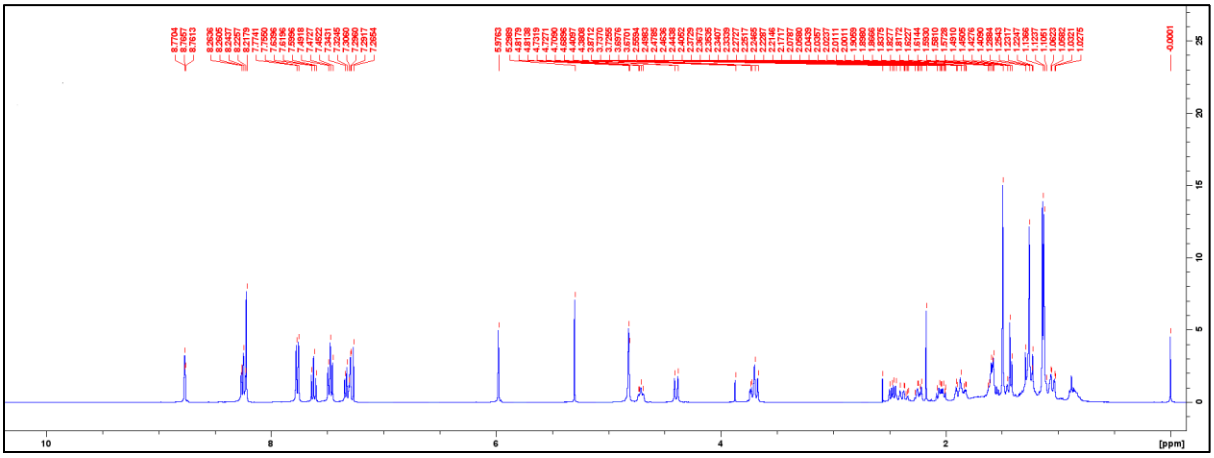

Figure 34SI: ^{1}H NMR spectrum of **2b**

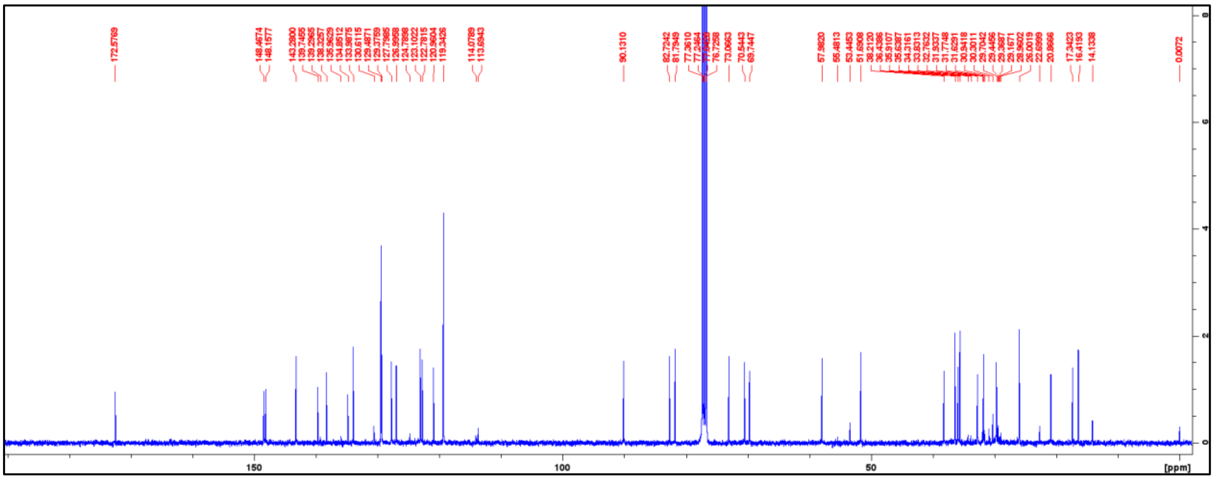

Figure 35SI: ^{13}C NMR spectrum of **2b**

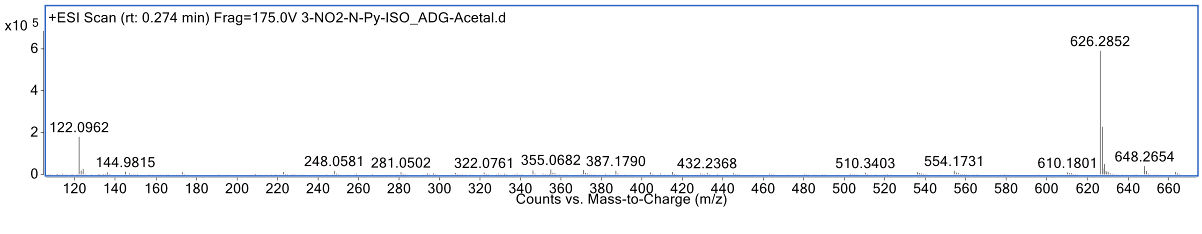

Figure 36SI: HRMS of **2b**

3,19-(N-phenyl-3-(3-fluorophenyl)-pyrazole) acetal of isoandrographolide (2c):

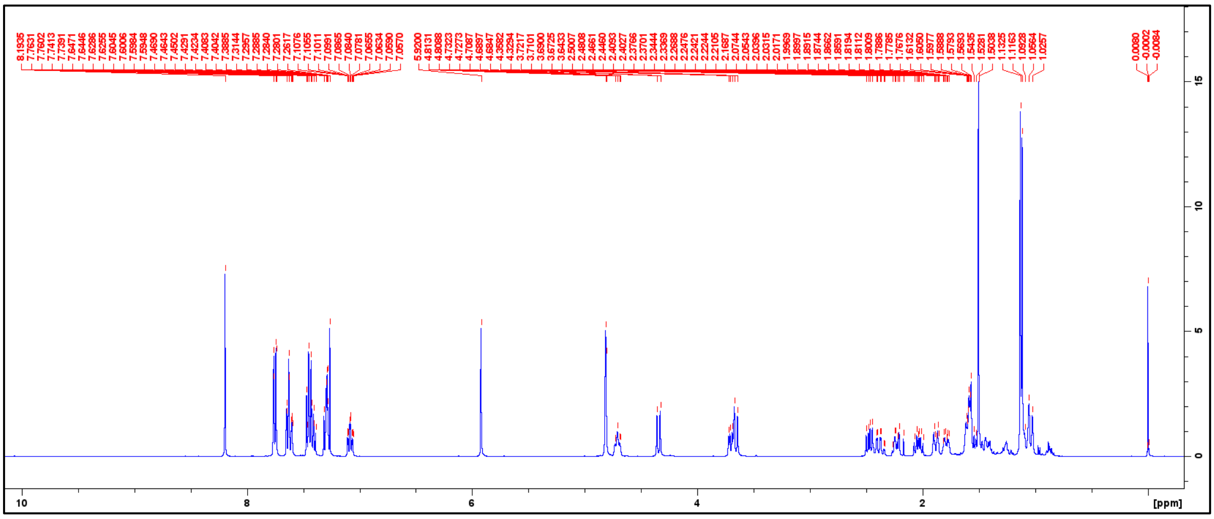

Figure 37SI: ^{1}H NMR spectrum of **2c**

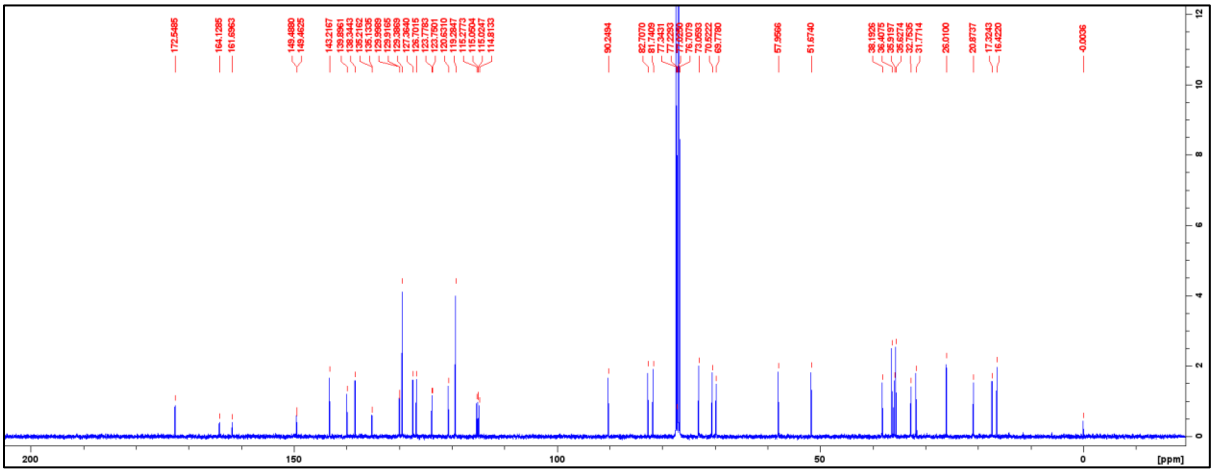

Figure 38SI: ^{13}C NMR spectrum of **2c**

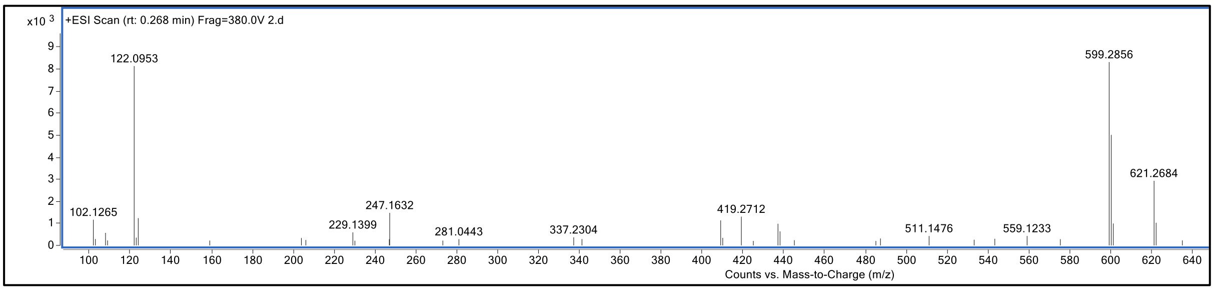

Figure 39SI: HRMS of **2c**

3,19-(N-phenyl-3-(3-chlorophenyl)-pyrazole) acetal of isoandrographolide (2d):

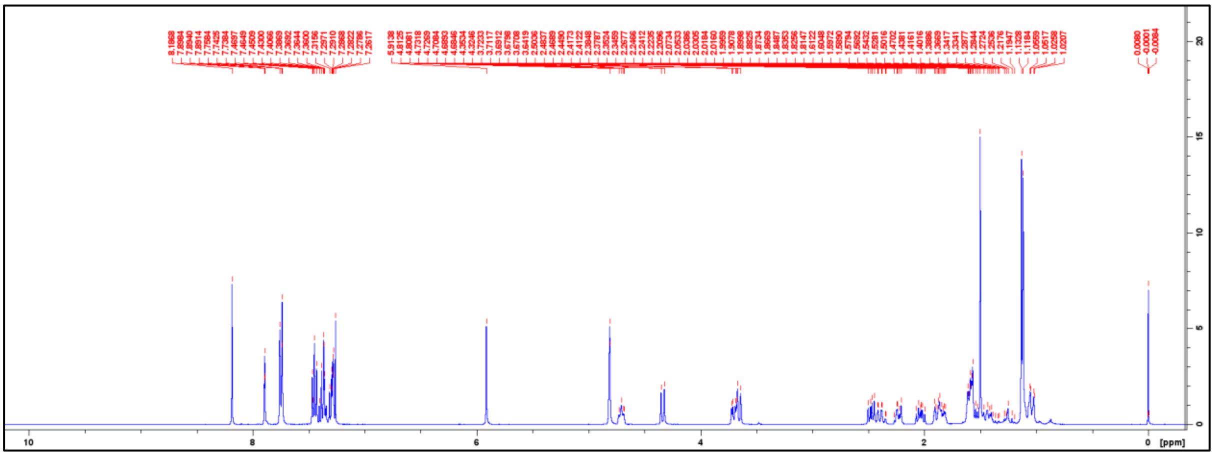

Figure 40SI: ^{1}H NMR spectrum of **2d**

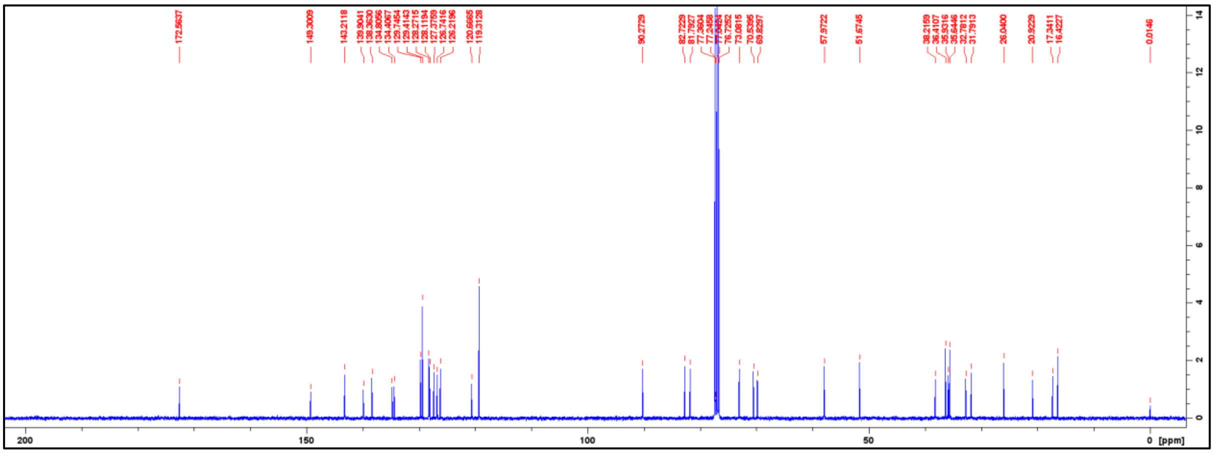

Figure 41SI: ^{13}C NMR spectrum of **2d**

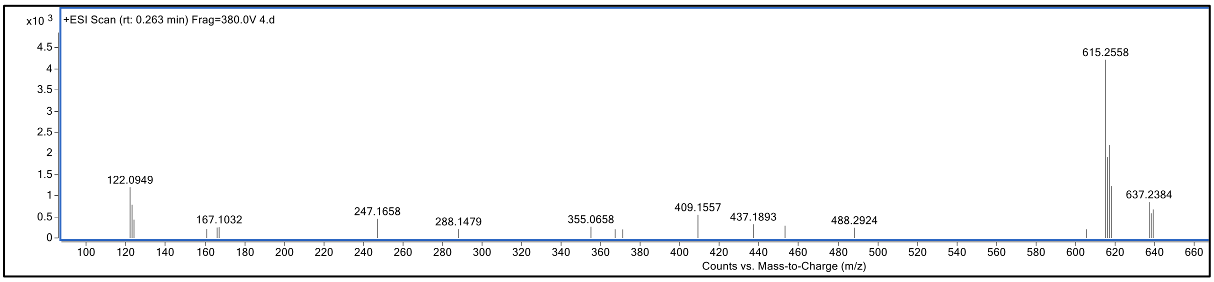

Figure 42SI: HRMS of **2d**

3,19-(N-phenyl-3-(3-bromophenyl)-pyrazole) acetal of isoandrographolide (2e)

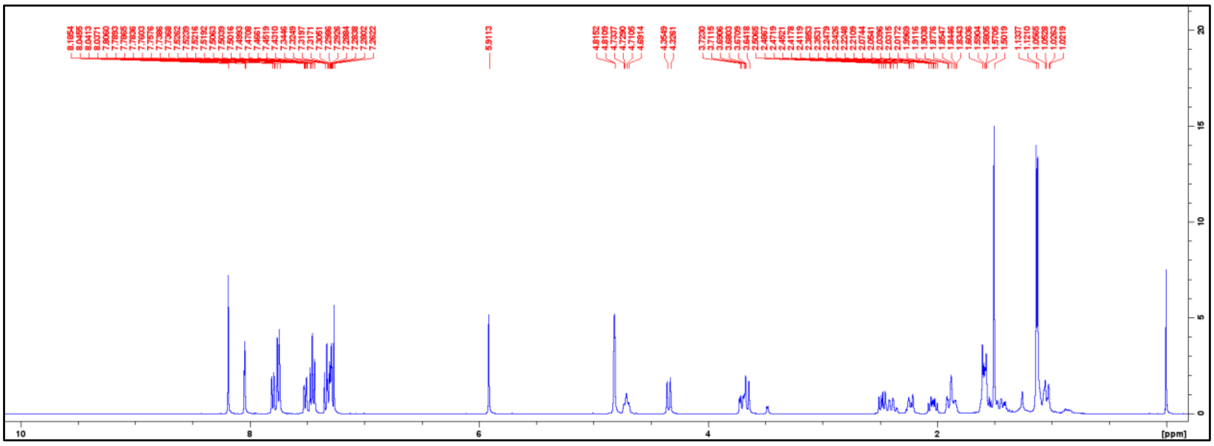

Figure 43SI: ^{1}H NMR spectrum of **2e**

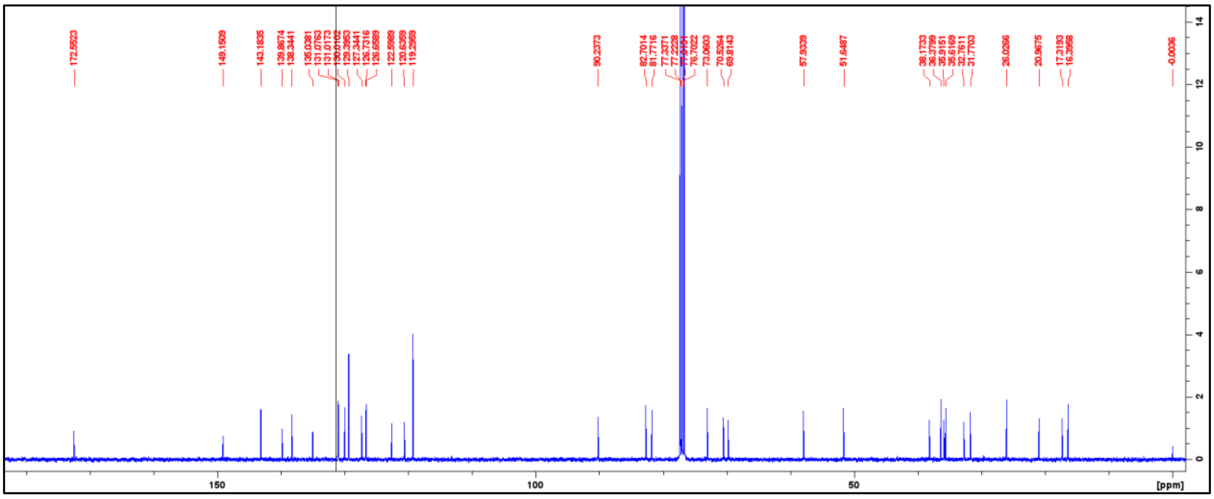

Figure 44SI: ^{13}C NMR spectrum of **2e**

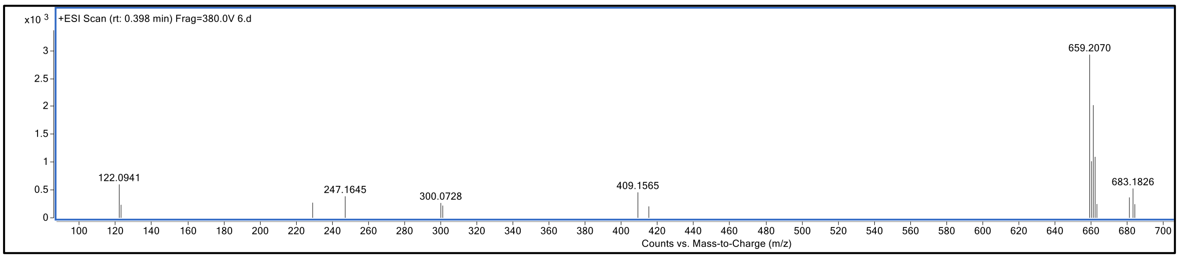

Figure 45SI: HRMS of **2e**

3,19-(N-phenyl-3-(4-fluorophenyl)-pyrazole) acetal of isoandrographolide (2f):

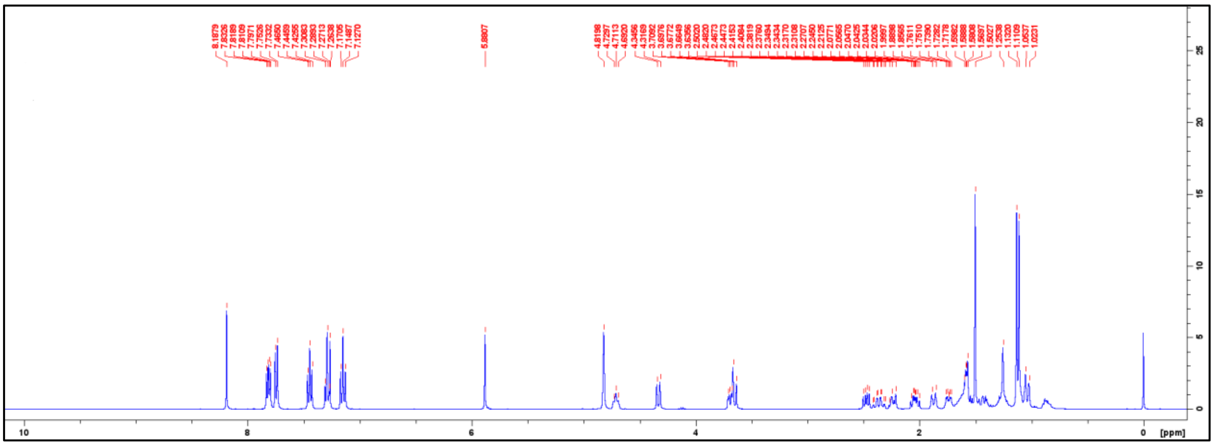

Figure 46SI: ^{1}H NMR spectrum of **2f**

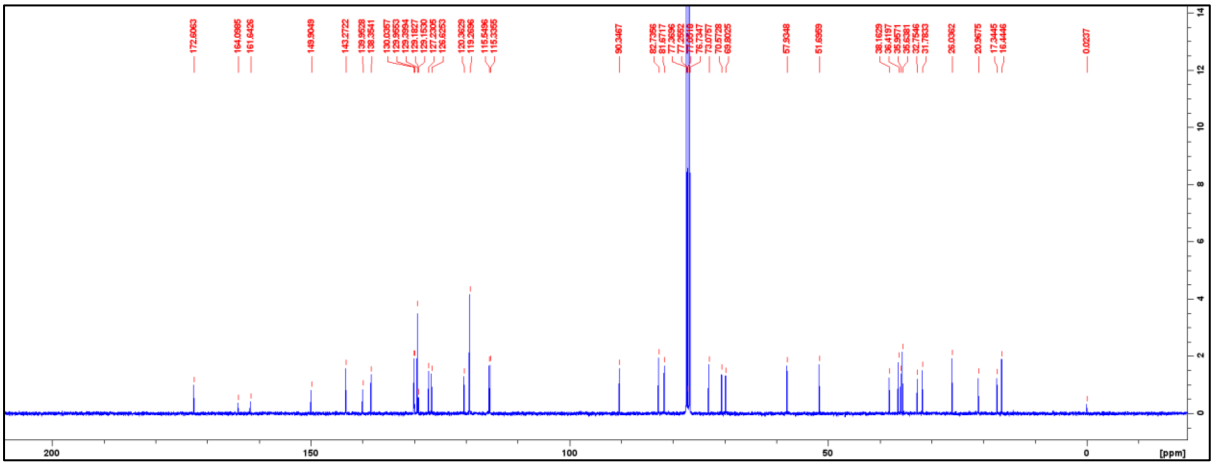

Figure 47SI: ^{13}C NMR spectrum of **2f**

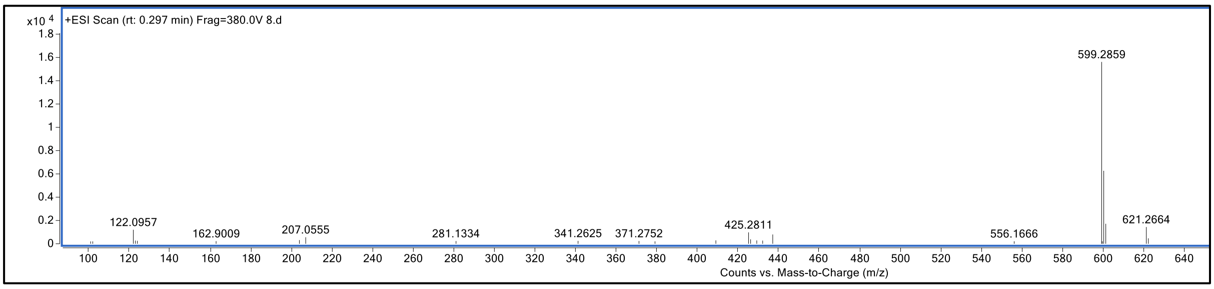

Figure 48SI: HRMS of **2f**

3,19-(N-phenyl-3-(4-chlorophenyl)-pyrazole) acetal of isoandrographolide (2g):

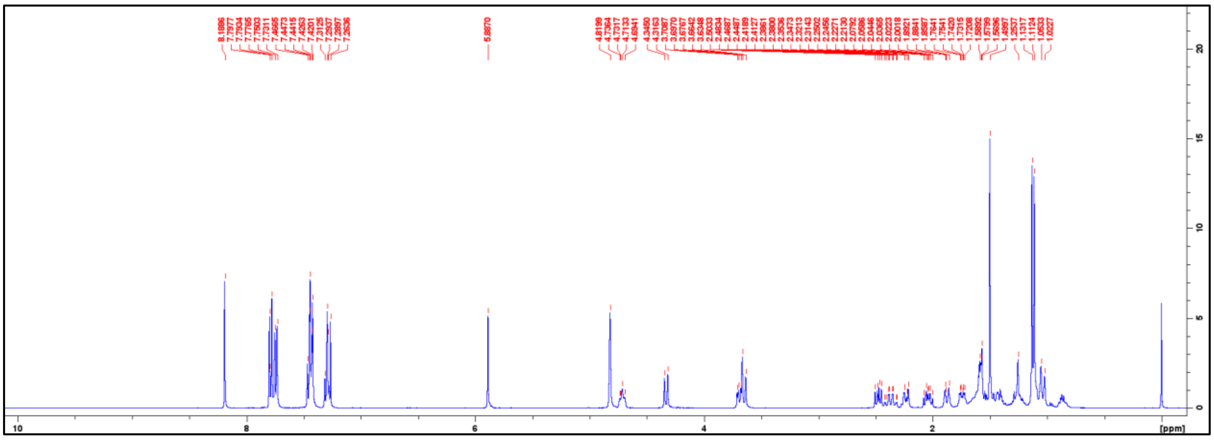

Figure 49SI: ^{1}H NMR spectrum of **2g**

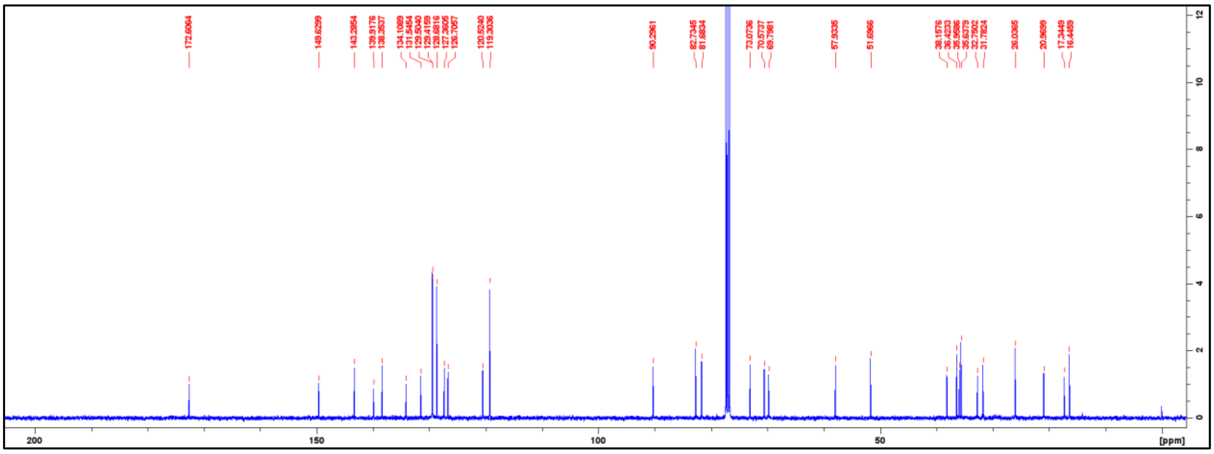

Figure 50SI: ^{13}C NMR spectrum of **2g**

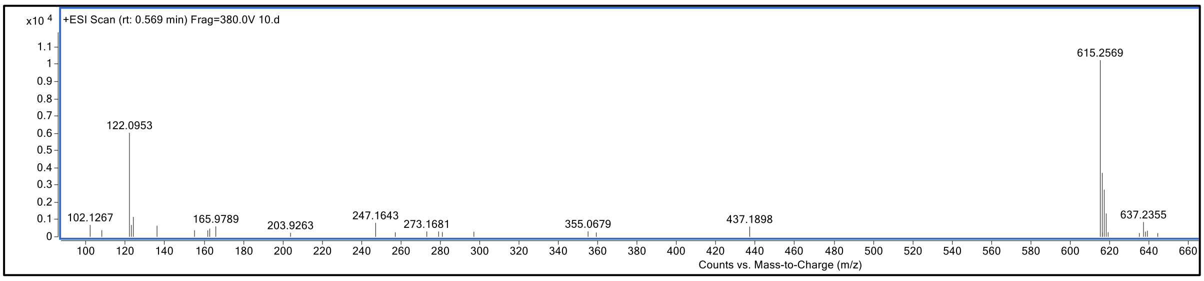

Figure 51SI: HRMS of **2g**

3,19-(N-phenyl-3-(4-bromophenyl)-pyrazole) acetal of isoandrographolide (2h):

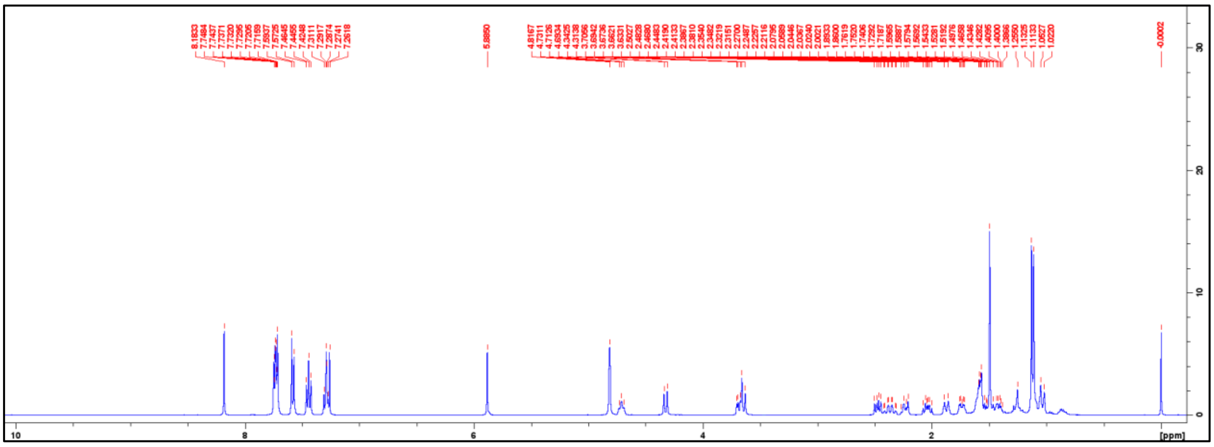

Figure 52SI: ^{1}H NMR spectrum of **2h**

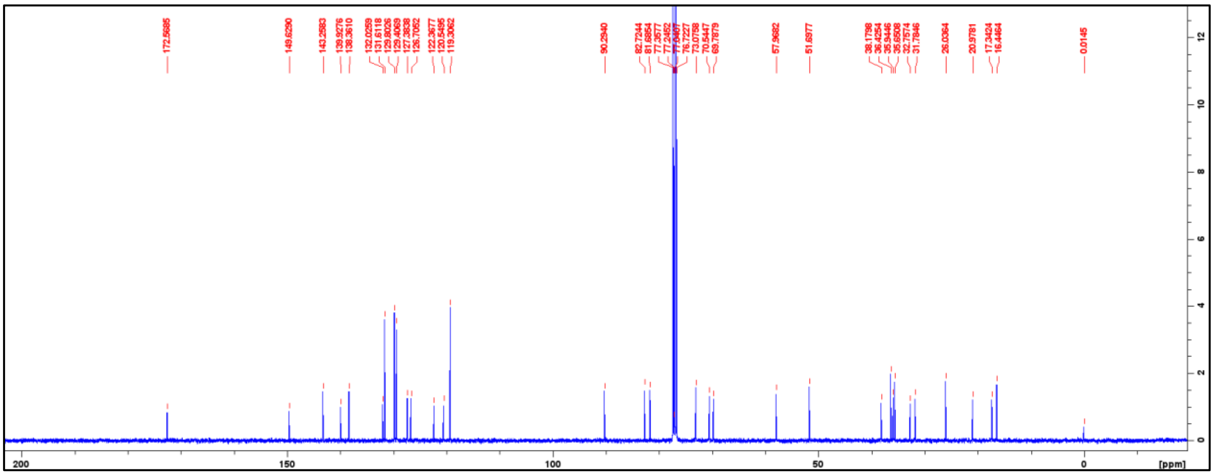

Figure 53SI: ^{13}C NMR spectrum of **2h**

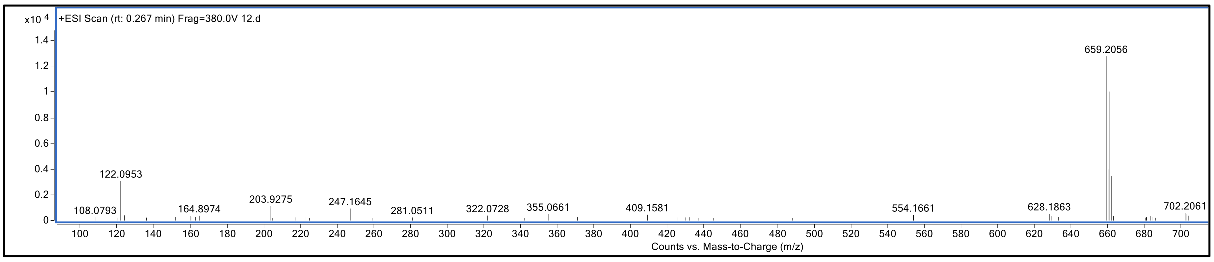

Figure 54SI: HRMS of **2h**

3,19-(N-phenyl-3-(4-methylphenyl)-pyrazole) acetal of isoandrographolide (2i)

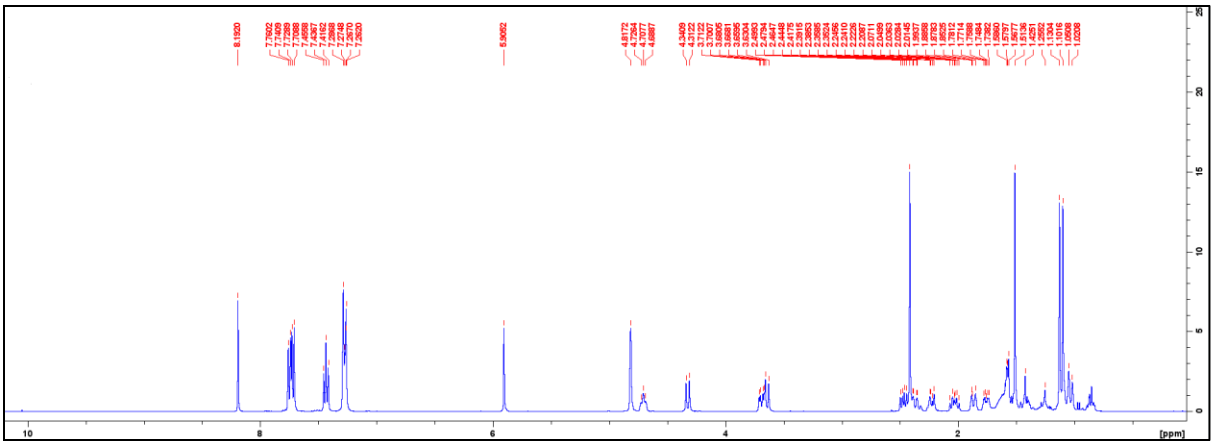

Figure 55SI: ^{1}H NMR spectrum of **2i**

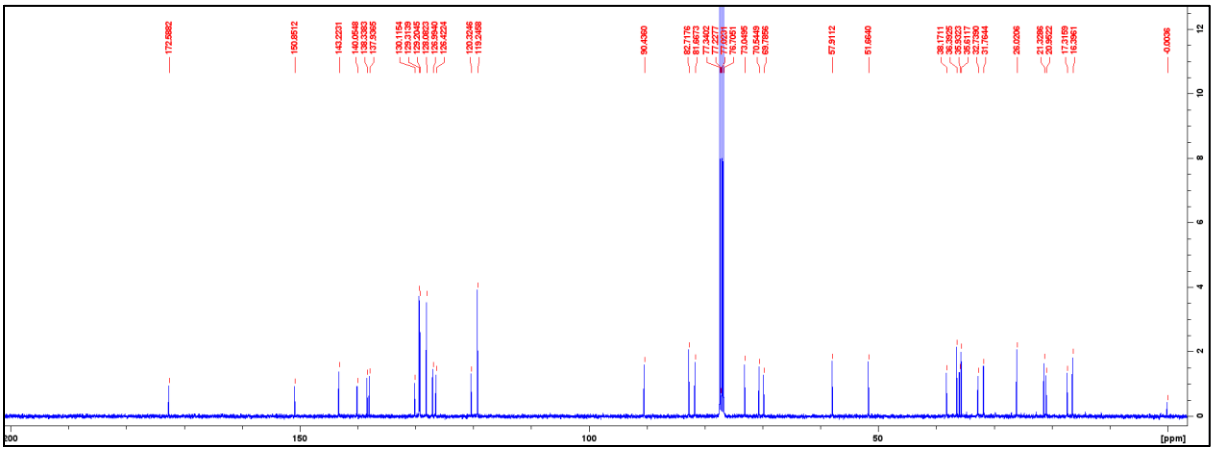

Figure 56SI: ^{13}C NMR spectrum of **2i**

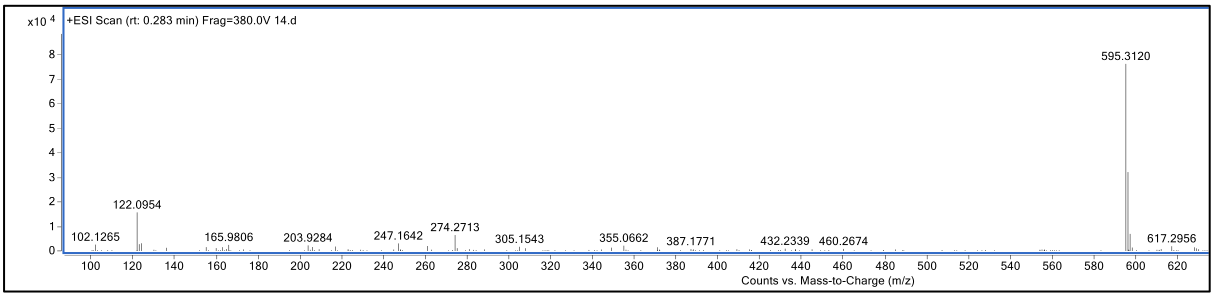

Figure 57SI: HRMS of **2i**

3,19-(N-phenyl-3-(4-methoxyphenyl)-pyrazole) acetal of isoandrographolide (2j)

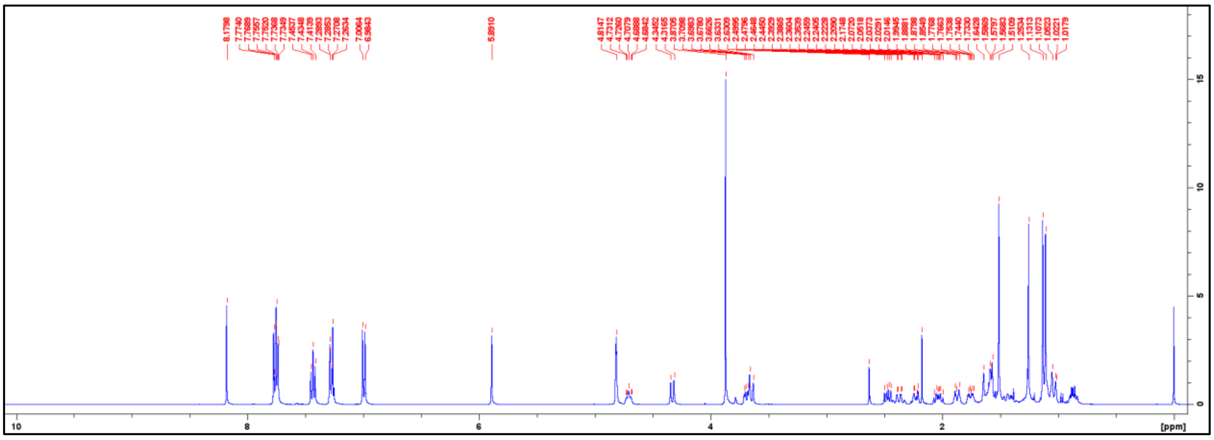

Figure 58SI: ^{1}H NMR spectrum of **2j**

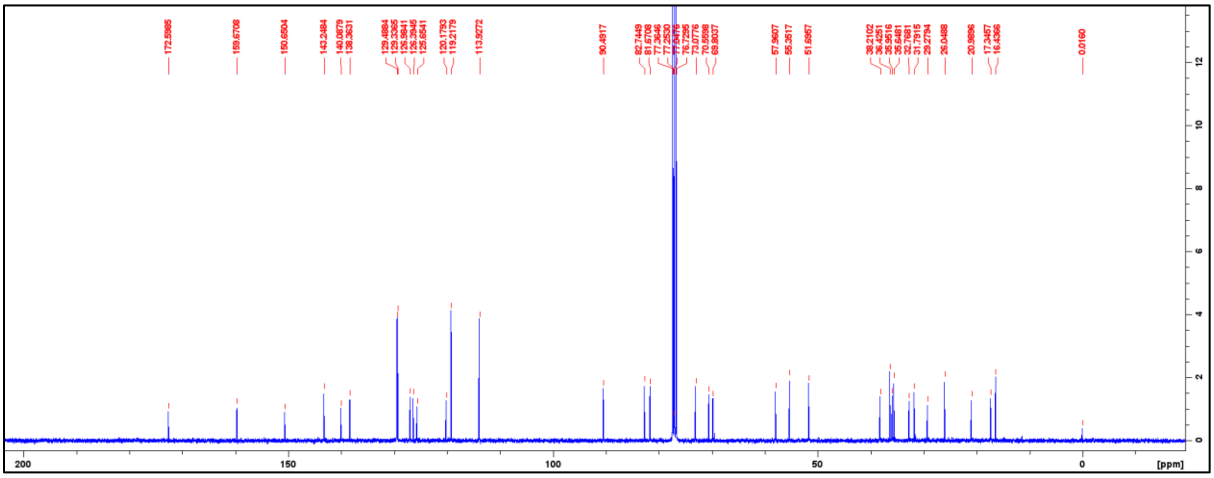

Figure 59SI: ^{13}C NMR spectrum of **2j**

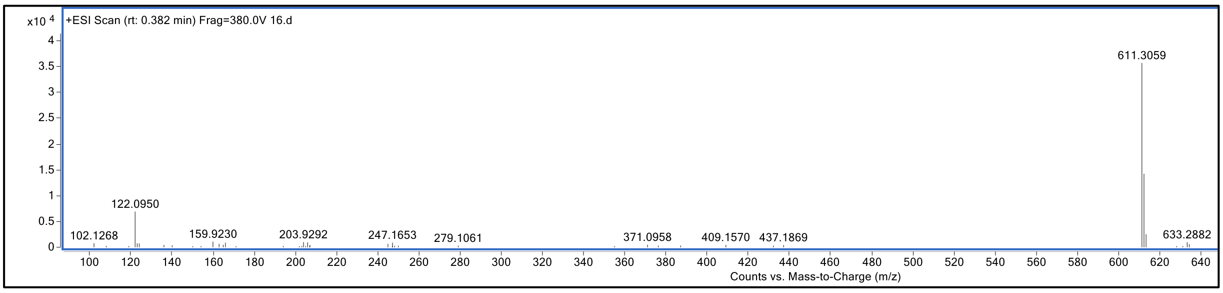

Figure 60SI: HRMS of **2j**

Table 1SI: Total growth inhibition (TGI) by the compounds **1a-1e** against NCI human cancer cell line panel.

Cancer	Sub Panel	TGI (μM)				
		**1a**	**1b**	**1c**	**1d**	**1e**
Leukemia	CCRF-CEM	3.37	5.01	> 100	16.30	NT
	HL-60(TB)	7.40	> 100	> 100	4.97	5.17
	K-562	> 100	> 100	> 100	NT	> 100
	MOLT-4	> 100	> 100	NT	3.82	4.24
	RPMI-8226	8.62	> 100	> 100	> 100	> 100
	SR	> 100	> 100	> 100	4.03	NT
Non-Small Cell Lung	A549/ATCC	7.11	> 100	25.30	11.30	19.90
	EKVX	4.66	7.98	5.17	4.95	5.16
	HOP-62	3.84	12.40	3.86	4.29	4.94
	HOP-92	2.99	3.64	3.03	3.11	3.41
	NCI-H226	4.31	6.59	3.19	3.37	3.43
	NCI-H23	3.08	3.55	3.57	3.82	4.07
	NCI-H322M	14.0	> 100	19.40	19.90	2.78
	NCI-H460	3.35	4.80	3.59	3.34	3.49
	NCI-H522	3.86	3.41	3.08	2.95	2.90
Colon	COLO 205	3.57	5.06	4.65	4.24	4.36
	HCC-2998	3.11	3.53	3.20	3.37	3.33
	HCT-116	3.55	4.25	1.59	3.48	2.75
	HCT-15	3.45	3.58	3.18	3.47	3.55
	HT29	6.77	4.26	4.51	3.13	3.46
	KM12	4.31	4.38	4.47	3.32	3.58
	SW-620	3.68	5.13	> 100	3.40	3.64
CNS	SF-268	20.50	> 100	5.61	3.34	4.26
	SF-295	3.05	3.19	2.88	2.75	2.68
	SF-539	3.26	3.18	3.17	3.02	3.20
	SNB-19	3.78	15.00	2.72	6.04	5.24
	SNB-75	3.90	50.01	3.55	3.14	5.32
	U251	3.71	3.35	5.61	3.00	3.13
Melanoma	LOX IMVI	3.00	3.34	2.75	2.97	3.03
	MALME-3M	3.18	3.55	3.50	2.85	3.45
	M14	3.62	4.52	3.59	3.88	4.12
	MDA-MB-435	4.19	18.30	3.08	3.19	3.60
	SK-MEL-2	3.54	3.51	3.29	3.05	3.38
	SK-MEL-28	3.38	4.06	3.35	3.37	3.28
	SK-MEL-5	2.70	3.00	3.02	3.00	2.88
	UACC-257	3.80	3.68	3.87	3.57	3.45
	UACC-62	3.20	3.46	3.21	3.42	3.40
Ovarian	IGROV1	3.44	4.27	5.72	3.22	4.83
	OVCAR-3	3.35	3.75	3.68	3.09	3.21
	OVCAR-4	NT	> 100	4.27	NT	NT
	OVCAR-5	NT	5.05	4.61	4.21	4.35
	OVCAR-8	4.02	5.04	3.88	3.44	4.22
	NCI/ADR-RES	5.31	NT	3.99	4.77	5.37
	SK-OV-3	18.70	> 100	6.09	33.40	48.30
Renal	786-0	4.47	10.30	3.59	6.10	NT
	A498	6.47	45.40	18.40	11.40	8.10
	ACHN	3.05	3.62	3.53	3.01	16.80
	CAKI-1	4.81	> 100	3.09	2.66	2.98
	RXF 393	2.84	3.20	3.12	2.31	2.93

	SN12C	3.96	10.30	3.42	3.62	2.55
	TK-10	3.39	5.82	NT	4.56	3.58
	UO-31	2.69	5.46	2.77	2.71	5.34
Prostate	PC-3	4.70	9.04	4.56	NT	NT
	DU-145	4.16	24.30	4.25	3.98	4.57
Breast	MCF7	9.09	> 100	5.49	66.80	> 100
	MDA-MB-231/ATCC	3.25	3.44	3.29	2.99	3.23
	HS 578T	6.54	NT	3.18	4.85	5.19
	BT-549	3.16	3.80	3.01	3.63	2.99
	T-47D	3.47	5.02	4.07	4.76	7.26
	MDA-MB-468	3.34	3.52	3.13	3.31	3.41

Table 2SI: Total growth inhibition (TGI) by the compounds **1f-1j** against NCI human cancer cell line panel.

Cancer	Sub Panel	TGI (μM)				
		1f	1g	1h	1i	1j
Leukemia	CCRF-CEM	NT	> 100	NT	> 100	> 100
	HL-60(TB)	7.12	> 100	NT	> 100	3.96
	K-562	> 100	> 100	NT	> 100	NT
	MOLT-4	NT	> 100	NT	> 100	7.57
	RPMI-8226	> 100	> 100	> 100	> 100	> 100
	SR	7.33	> 100	8.13	> 100	5.21
Non-Small Cell Lung	A549/ATCC	5.92	36.30	51.60	13.50	7.29
	EKVX	4.72	5.28	5.35	4.96	4.49
	HOP-62	3.36	4.95	3.83	4.44	10.40
	HOP-92	2.73	3.08	2.92	3.14	3.02
	NCI-H226	2.94	3.05	2.80	2.95	3.36
	NCI-H23	3.32	3.59	3.62	3.50	3.74
	NCI-H322M	15.80	36.50	26.40	25.30	16.50
	NCI-H460	3.33	3.72	3.34	3.56	3.50
	NCI-H522	3.82	3.12	3.37	3.46	3.24
Colon	COLO 205	3.20	5.58	3.51	4.50	4.24
	HCC-2998	3.21	3.39	3.49	3.06	3.19
	HCT-116	1.18	2.53	2.48	1.52	3.11
	HCT-15	3.18	3.21	3.30	3.19	3.37
	HT29	7.63	4.64	6.51	4.58	3.38
	KM12	3.59	4.92	3.68	7.93	3.31
	SW-620	> 100	> 100	> 100	NT	3.80
CNS	SF-268	5.63	6.32	4.68	7.58	3.60
	SF-295	2.92	2.88	2.93	2.96	2.78
	SF-539	3.16	3.12	3.18	3.19	3.10
	SNB-19	2.91	NT	NT	NT	7.42
	SNB-75	3.09	2.87	2.91	2.99	5.09
	U251	5.63	3.19	2.89	3.12	3.10
Melanoma	LOX IMVI	2.59	2.94	2.90	2.74	2.93
	MALME-3M	3.02	3.41	3.11	4.28	2.93
	M14	3.23	3.69	3.55	3.61	3.54
	MDA-MB-435	3.27	3.28	3.34	3.31	3.17
	SK-MEL-2	3.10	3.25	3.18	3.28	3.08
	SK-MEL-28	3.12	3.36	3.30	3.33	3.69
	SK-MEL-5	2.98	3.11	2.96	2.96	2.89
	UACC-257	3.18	3.63	3.35	3.47	3.28

	UACC-62	3.09	3.27	3.13	3.15	4.26
Ovarian	IGROV1	4.75	46.10	5.16	12.50	2.99
	OVCAR-3	3.71	3.72	3.32	3.82	2.96
	OVCAR-4	4.08	5.15	4.27	4.27	4.23
	OVCAR-5	3.88	5.38	5.08	4.59	4.21
	OVCAR-8	2.95	4.97	3.40	3.51	2.84
	NCI/ADR-RES	3.57	4.24	4.19	3.71	4.41
	SK-OV-3	5.48	1.67	8.27	12.10	23.90
Renal	786-0	3.34	4.17	4.49	3.69	5.59
	A498	11.50	19.00	1.66	15.80	11.30
	ACHN	3.11	3.95	4.78	3.50	2.87
	CAKI-1	3.17	3.28	3.49	3.37	2.64
	RXF 393	3.04	3.37	3.23	2.97	2.20
	SN12C	3.27	3.57	3.37	3.44	5.94
	TK-10	2.55	2.74	NT	NT	4.13
	UO-31	3.34	4.17	2.52	2.67	2.54
Prostate	PC-3	4.04	> 100	NT	NT	NT
	DU-145	3.44	4.10	3.59	4.19	4.94
Breast	MCF7	4.13	> 100	4.81	4.60	> 100
	MDA-MB-231/ATCC	3.28	4.60	3.14	3.28	2.98
	HS 578T	2.94	3.24	3.01	3.41	4.88
	BT-549	3.18	3.37	3.50	3.02	3.23
	T-47D	2.97	3.60	3.13	3.55	5.02
	MDA-MB-468	2.96	4.49	3.47	3.45	3.25

Table 3SI: 50% lethal concentration (LC_{50}) by the compounds **1a-1e** against NCI human cancer cell line panel.

**Cancer**	**Sub Panel**	**LC_{50} (μM)**				
		**1a**	**1b**	**1c**	**1d**	**1e**
Leukemia	CCRF-CEM	> 100	> 100	> 100	> 100	> 100
	HL-60(TB)	> 100	> 100	> 100	> 100	> 100
	K-562	> 100	> 100	> 100	> 100	> 100
	MOLT-4	> 100	> 100	> 100	> 100	> 100
	RPMI-8226	> 100	> 100	> 100	> 100	> 100
	SR	> 100	> 100	> 100	> 100	> 100
Non-Small Cell Lung	A549/ATCC	> 100	> 100	> 100	69.10	> 100
	EKVX	> 100	> 100	56.00	> 100	> 100
	HOP-62	7.89	> 100	8.55	> 100	> 100
	HOP-92	5.83	7.11	6.02	6.20	NT
	NCI-H226	9.55	> 100	6.08	6.46	6.71
	NCI-H23	5.64	6.87	7.09	8.31	8.74
	NCI-H322M	83.70	> 100	> 100	> 100	> 100
	NCI-H460	6.74	11.30	3.59	6.66	6.83
	NCI-H522	7.85	6.65	6.24	5.65	5.62
Colon	COLO 205	6.31	12.70	> 100	9.07	NT
	HCC-2998	5.76	6.48	5.92	6.31	6.41
	HCT-116	7.83	NT	4.40	45.20	NT
	HCT-15	6.91	7.37	6.45	7.64	7.85
	HT29	> 100	9.95	> 100	6.57	7.23
	KM12	4.31	NT	4.47	6.61	7.01
	SW-620	7.82	> 100	> 100	7.55	NT
CNS	SF-268	> 100	> 100	28.40	6.94	8.98
	SF-295	5.67	6.05	5.49	5.36	5.30

	SF-539	5.77	5.79	5.68	5.57	5.74
	SNB-19	7.73	60.00	NT	54.80	17.20
	SNB-75	26.50	> 100	5.82	8.84	22.90
	U251	7.48	6.46	6.99	5.69	5.81
Melanoma	LOX IMVI	5.85	6.48	5.25	5.50	5.59
	MALME-3M	5.88	7.05	6.55	5.52	6.36
	M14	7.23	9.85	7.40	8.96	NT
	MDA-MB-435	9.43	> 100	6.32	6.49	7.51
	SK-MEL-2	7.20	6.94	6.02	5.91	6.45
	SK-MEL-28	6.45	8.11	6.36	6.33	6.20
	SK-MEL-5	5.20	5.53	5.51	5.53	5.39
	UACC-257	7.62	7.53	7.78	7.20	6.72
	UACC-62	6.22	7.06	6.22	6.83	6.90
Ovarian	IGROV1	6.66	8.76	> 100	7.50	> 100
	OVCAR-3	6.16	7.55	6.94	5.95	6.08
	OVCAR-4	> 100	> 100	> 100	> 100	> 100
	OVCAR-5	8.71	NT	31.00	7.73	47.70
	OVCAR-8	> 100	> 100	8.50	> 100	> 100
	NCI/ADR-RES	NT	NT	8.99	> 100	> 100
	SK-OV-3	91.10	> 100	> 100	> 100	> 100
Renal	786-0	9.83	> 100	7.24	83.40	> 100
	A498	27.70	> 100	53.30	38.10	44.0
	ACHN	5.67	6.94	6.94	5.52	5.51
	CAKI-1	NT	> 100	6.13	5.39	6.04
	RXF 393	5.76	6.32	5.94	5.07	5.51
	SN12C	8.37	> 100	7.34	7.92	8.12
	TK-10	6.46	> 100	NT	9.71	40.40
	UO-31	5.36	35.70	5.46	5.63	6.83
Prostate	PC-3	66.90	> 100	> 100	> 100	> 100
	DU-145	NT	> 100	9.15	8.43	9.82
Breast	MCF7	> 100	> 100	50.10	> 100	> 100
	MDA-MB-231/ATCC	6.15	6.44	6.28	5.97	6.23
	HS 578T	> 100	> 100	6.12	> 100	> 100
	BT-549	6.10	NT	NT	NT	NT
	T-47D	8.47	> 100	> 100	> 100	> 100
	MDA-MB-468	6.58	7.00	6.01	6.30	6.37

Table 4SI: 50% lethal concentration (LC_{50}) by the compounds **1f-1j** against NCI human cancer cell line panel.

Cancer	Sub Panel	LC_{50} (μM)				
		**1f**	**1g**	**1h**	**1i**	**1j**
Leukemia	CCRF-CEM	> 100	> 100	> 100	> 100	> 100
	HL-60(TB)	> 100	> 100	> 100	> 100	> 100
	K-562	> 100	> 100	> 100	> 100	> 100
	MOLT-4	> 100	> 100	> 100	> 100	> 100
	RPMI-8226	> 100	> 100	> 100	> 100	> 100
	SR	> 100	> 100	> 100	> 100	> 100
Non-Small Cell Lung	A549/ATCC	51.50	> 100	> 100	> 100	62.20
	EKVX	> 100	> 100	> 100	> 100	> 100
	HOP-62	6.95	> 100	8.12	9.27	86.30
	HOP-92	5.78	6.32	6.20	6.88	6.20
	NCI-H226	5.75	5.99	5.77	5.92	6.55

	NCI-H23	6.46	7.45	7.67	6.96	8.20
	NCI-H322M	90.60	36.50	> 100	> 100	> 100
	NCI-H460	6.57	7.76	7.00	6.91	7.01
	NCI-H522	7.48	6.17	6.68	6.86	6.06
Colon	COLO 205	6.92	> 100	7.24	13.70	NT
	HCC-2998	5.86	6.14	6.64	5.76	5.98
	HCT-116	NT	5.37	6.30	4.65	NT
	HCT-15	6.28	6.51	6.83	6.38	7.20
	HT29	93.90	> 100	> 100	> 100	6.73
	KM12	7.02	> 100	8.20	76.90	6.63
	SW-620	> 100	> 100	> 100	> 100	8.55
CNS	SF-268	25.70	> 100	11.50	55.40	7.77
	SF-295	5.49	5.51	5.57	5.54	5.39
	SF-539	5.65	5.66	5.73	5.70	5.70
	SNB-19	NT	NT	NT	NT	34.60
	SNB-75	5.86	5.87	5.75	6.55	24.40
	U251	5.75	6.00	5.51	5.70	5.78
Melanoma	LOX IMVI	5.09	5.48	5.41	5.25	5.47
	MALME-3M	5.76	6.41	6.02	7.45	5.59
	M14	NT	7.38	7.36	7.38	7.27
	MDA-MB-435	6.38	6.51	6.67	6.79	6.51
	SK-MEL-2	5.70	5.97	5.77	6.01	6.02
	SK-MEL-28	5.86	6.35	6.30	6.39	7.01
	SK-MEL-5	5.47	5.69	5.44	5.49	5.41
	UACC-257	6.11	7.32	6.63	6.95	6.56
	UACC-62	6.05	6.39	6.20	6.04	9.48
Ovarian	IGROV1	> 100	> 100	> 100	> 100	6.52
	OVCAR-3	6.79	7.77	7.25	7.49	5.84
	OVCAR-4	NT	> 100	> 100	NT	> 100
	OVCAR-5	8.66	> 100	> 100	40.50	9.52
	OVCAR-8	5.86	> 100	7.45	7.37	5.97
	NCI/ADR-RES	8.29	9.87	NT	8.34	> 100
	SK-OV-3	30.70	> 100	> 100	> 100	> 100
Renal	786-0	6.96	8.70	9.76	3.34	> 100
	A498	38.30	53.70	53.60	48.80	39.20
	ACHN	5.82	8.23	> 100	6.67	5.41
	CAKI-1	6.34	6.59	7.69	6.64	5.54
	RXF 393	5.78	6.33	6.11	5.92	4.98
	SN12C	6.93	7.61	7.48	7.15	65.10
	TK-10	NT	NT	NT	NT	8.77
	UO-31	5.14	5.60	5.34	5.36	5.25
Prostate	PC-3	> 100	> 100	> 100	> 100	> 100
	DU-145	6.52	NT	7.28	8.55	16.40
Breast	MCF7	63.10	42.80	72.00	5.40	> 100
	MDA-MB-231/ATCC	6.41	6.25	6.19	6.33	60.10
	HS 578T	5.72	6.45	5.94	6.28	> 100
	BT-549	NT	NT	3.50	NT	NT
	T-47D	7.44	> 100	8.33	> 100	> 100
	MDA-MB-468	5.71	6.36	6.23	6.43	6.16
